# Transient frontopolar cortex stimulation induces prolonged disruption to counterfactual processing

**DOI:** 10.1371/journal.pbio.3003495

**Published:** 2025-11-18

**Authors:** Matthew Ainsworth, Juan M. Galeazzi, Carlos Pedreira, Mark G. Stokes, Mark J. Buckley

**Affiliations:** Department of Experimental Psychology, University of Oxford, Life and Mind Building, Oxford, United Kingdom; National Institute on Drug Abuse Intramural Research Program, UNITED STATES OF AMERICA

## Abstract

Frontopolar cortex (FPC) contains area 10, an anterior sub-region of prefrontal cortex exclusive to humans and nonhuman primates (NHPs) which is thought to support monitoring the value of switching between alternative goals. However, the neuronal mechanisms underlying this function are unclear. Here, we used multielectrode arrays to record the local field potentials (LFPs) in the FPC of two macaques performing a Wisconsin Card Sorting Test analogue and found that bursts of gamma and beta in FPC tracked counterfactual not current rule value. Moreover, we show that brief high-frequency microstimulation to a single trial causally affects both LFP activity in FPC, as well as rule-guided decision-making across successive trials. Following stimulation of FPC, we observed reduced exploration of the counterfactual rule prerule-change, as well as a delayed adaptation to the newly relevant rule postrule-change. A similar, multi-trial time-course disturbance to beta and gamma activity within FPC was also induced following single-trial microstimulation. These findings link neuronal activity in FPC with behavioral monitoring of the value of counterfactual rules and provide neural mechanistic insights into how FPC supports rule-based decision-making.

## Abstract 2

What role does the frontopolar cortex (FPC) play in evaluating alternative goals? This study shows that FPC activity tracks the value of counterfactual rules through gamma and beta bursts, and that targeted FPC stimulation alters both neural signals and decision-making, revealing a causal link between FPC dynamics and rule-based behavioral flexibility.

## Introduction

Frontopolar cortex (FPC) is occupied by area 10 and is one of the larger sub-regions of primate prefrontal cortex [1], but our understanding of its contributions to cognition remains nascent. Despite often being regarded as an area supporting some of the highest-order cognitive processes in humans including reasoning, planning, and abstraction, no clear consensus has emerged as to its precise role or mechanistic contribution. One recent proposal that emerged from considering both the human and nonhuman primate (NHP) literature is that FPC influences cognitive flexibility, maintaining an appropriate balance between exploration and exploitation, evaluating the relative value of alternatives, and monitoring competing goals [2]. This contribution of primate FPC to cognition may be distinct from that of other posterior prefrontal regions as supported by a number of correlative findings from both human neuroimaging [3–6] as well as causal findings from circumscribed FPC lesion behavioral studies in macaque [7–10]. Damage to FPC decreases behavioral flexibility in both humans and NHPs. Patients whose lesions encroach substantially upon FPC are more impaired at multi-tasking [11], task-switching [12], and prospective memory-based task-selection [13], than patients whose lesions do not encroach so extensively upon FPC. Moreover, the aforementioned exploit/explore trade-off can also explain observations of (otherwise counterintuitive) FPC lesion-induced performance enhancements in certain situations. For example, patients whose rostral prefrontal lesions encompassed FPC outperformed nonlesioned controls when required to keep exploiting the previous rule [12]. Similarly, macaques with circumscribed FPC lesions remained well focused on a complex primary task and continued to effectively exploit rewards therein despite distractions, significantly outperforming normal controls with intact FPC whose performance dropped to chance with the same distractions [9].

Nonetheless, our understanding of the specific contribution of FPC to cognition is stymied by a paucity of neuronal data [14]. There are no published reports of neuronal activity from electrodes implanted within human FPC, and only limited recordings from macaque FPC. Initial macaque studies highlighted involvement of FPC in monitoring self-generated decisions [15*–*17]. More recent electrophysiological recordings from FPC have demonstrated neurons which encode signals related to the monitoring of actions of other agents [18] or goals during fast learning [19]. Both findings are consistent with previous literature: several fMRI studies have shown activation of FPC when monitoring social interactions [20,21] and lesions of FPC have been shown to cause deficits in fast learning [7]. However, while these findings are consistent with the purported role for FPC in monitoring the relative benefits of exploitation versus exploration [2] the nature of the neuronal mechanisms in FPC that support these explore/exploit processes in general remains unclear. For example, nothing is known of the frequency-specific oscillatory dynamics within FPC, and the implications this activity has for information encoding, nor whether disruption of such activity in turn influences this brain region’s contribution to monitoring counterfactuals/alternatives.

In this study, we recorded the local field potentials (LFPs) from FPC of two monkeys (*Macaca mulatta*) while they performed an analogue of the Wisconsin Card Sort Task (WCST), a well-established behavioral task that involves abstract rule-guided decision-making and periodic reversals between choices based on the changing values of alternative uncued abstract rules (i.e., matching-by-color, and matching-by-shape) [9,22]. Using a combination of reinforcement learning (RL) modeling, recordings from arrays in FPC, and direct electrical microstimulation through electrodes in these arrays, we tested the causal contribution of FPC neuronal activity to rule-guided (and counterfactual rule-guided) decision-making.

We present mechanistic evidence linking beta and gamma frequency activity with counterfactual rule valuation, and exploratory behavior. We show that gamma frequency activity within FPC, aligned to the animals’ choices, tracked reward/feedback, while bursts of gamma and beta activity in FPC tracked the value of the counterfactual (to the currently reinforced rule). Furthermore, targeted causal manipulation of endogenous neuronal activity within FPC using electrical microstimulation significantly modified the amplitude of the LFP recorded from FPC as well as the animal’s performance in the task. Specifically, stimulation of FPC to a single trial about 10 trials before the next rule-change both decreased the exploration of the counterfactual rule during trials leading up to the uncued rule/block change, and also increased the animal’s perseverance after the rule change. These changes were accompanied by trial specific changes in beta and gamma activity in FPC. Together these findings provide further evidence linking FPC with control of exploratory behavior and provides the demonstration of a mechanistic link between LFP activity recorded from FPC and variables needed to guide exploratory behavior.

## Results

### Animals’ behavioral choices are influenced by the value of the counterfactual rule

**Fig 1:**
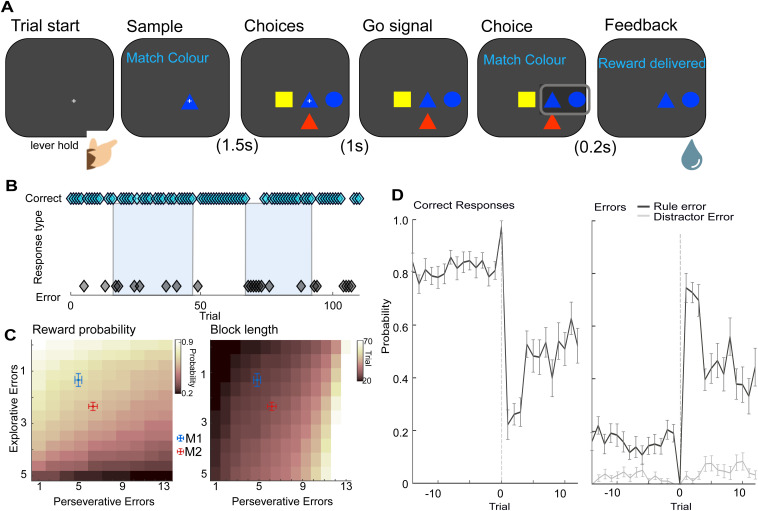
Outline of the WCST analogue and the monkeys’ behavioral performance A. Task schematic: monkeys matched a central sample stimulus-item to one of three surrounding choice-items based either on a color or shape-matching rule; the currently correct rule was uncued and changed on a block-by-block basis whenever they attained 85% correct in 20 consecutive trials. **B.** Behavioral performance during a single recording session. Trial outcome indicated with cyan (correct response, top) or black (rule errors, bottom) diamonds. Shape-matching block indicated by blue background and color-matching block by white. Note rule errors occur throughout blocks. **C**. Colormaps showing the idealized expected reward (*left*) and block length (*right*) relative to the number of explorative and perseverative errors. Cross-hairs show the animal’s actual performance (mean ± SE). **D.** Averaged performance of both animals, aligned to block changes (trial 0). Mean correct responses (*left*), and rule and distractor errors (*right*) shown ±SEM. Note consistent explorative rule errors prior to block change.

On each trial of the WCST analogue, the monkeys were required to select one of three choice stimuli, presented around a central sample stimulus, based on either the color or shape of that sample stimulus (Fig 1A and Materials and methods for further details). Of these three choice stimuli one matched the sample in color, one in shape, while the third distractor stimulus, matched none of the features of the sample. The correct rule to apply to gain reward on any given trial (i.e., to color-match or shape-match) was never cued to the animal and so had to be learnt by trial-and-error and then retained in memory across trials. The reinforced rule alternated without announcement between blocks whenever monkeys attained a performance criterion of 85% correct in 20 consecutive trials.

Both monkeys received extensive training on this task prior to this study. For recording-only sessions collected in this study, animals completed an average of 5 blocks per session (mean block/rule changes per session were 6.25 and 3.5 for M1 and M2, respectively). On average, there were 127 correct trials per recording session (151 and 102 correct trials/session for M1 and M2, respectively). Visualization of the correct and incorrect responses made by monkeys (Fig 1B and 1D) revealed several notable aspects of the animals’ behavior. The majority of incorrect responses made by both monkeys were “rule errors” in which the monkeys chose a stimulus corresponding to the incorrect/counterfactual rule (i.e., matching the shape rather than the color of a stimulus, or vice-versa). By contrast, the monkeys rarely chose the distractor stimulus which neither matched the sample in color nor shape (Fig 1D) indicating that on the vast majority of error trials they still know a matching rule applies even if they do not always implement the correct one.

The frequency with which the monkeys made rule errors varied markedly relative to rule changes. During the initial trials on each block (immediately after rule changes), monkeys made numerous rule errors as they perseverated with the previous rule (“perseverative errors”). However, the monkeys continued to make some rule errors throughout the block, including on the trials before a rule-switch when the correct rule to exploit was well established (“explorative errors”, Fig 1B and 1D). These explorative errors were made with comparable reaction times to correct choices (S1 Fig), suggesting that the animals planned such exploration early in trials (and hence avoided any potential reaction time penalty associated with switching [23]). Comparison of errors made by both animals with idealized models predicting reward delivery and block length (Fig 1C) relative to rule errors (both explorative and perseverative) revealed that neither animal used the optimum behavioral strategy (i.e., determining the current rule and exploiting it unwaveringly until the rule changes) to perform the task. Instead, both animals made a small number of explorative errors throughout testing sessions which avoided causing a significant behavioral penalty (either in terms of reward receipt or increased block length). These data suggest that both animals employed an alternative behavioral strategy in which they periodically tested the counterfactual rule throughout the block. Crucially, this strategy attaches a value not only to the rule associated with their choice but also with the unchosen rule, suggesting that the monkeys simultaneously maintained and updated the value for both the current (i.e., correct) and the counterfactual (i.e., incorrect) rule, with both updated in response to the trial outcome.

**Fig 2:**
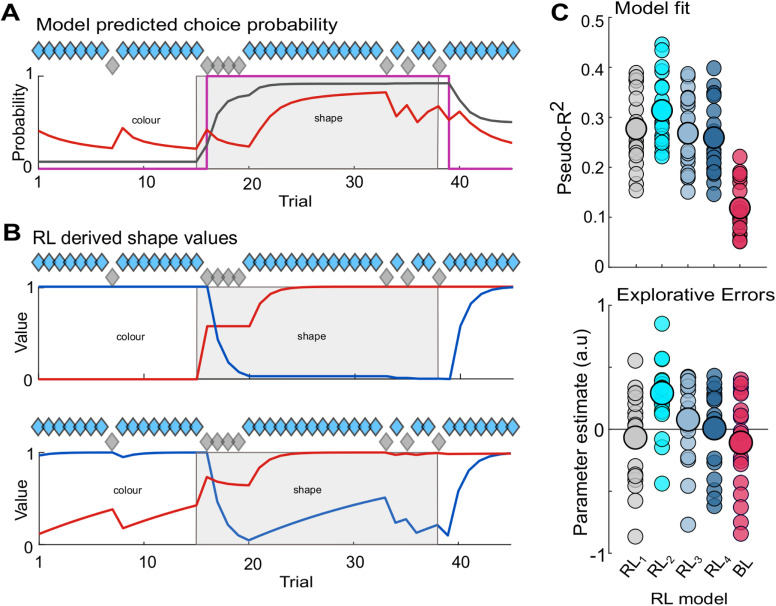
Behavioral modeling of monkeys’ WCST performance. **A.** Example data showing the model-derived probability of animals choosing the shape cue for the standard RL model (black RL_1 chosen_), a Bayesian learner (red), and for the ideal observer (fuscia). Diamonds denote correct/incorrect trials (blue/gray, respectively), shape block shown in light gray. **B.** RL derived expected values for the same data, showing the value of the shape (red) and color (blue) rules derived from the standard RL model (*upper*, RL_1 chosen_) and the modified model in which both the chosen and unchosen values are updated (*lower*, RL_2 chosen + unchosen_). Note the increases in expected value for counterfactual rules in RL_2 chosen + unchosen_. **C.** Model fit of defined as Mcfadden’s Pseudo *r*^2^ for four models (*upper*) *RL*_*1 chosen*_ (*gray*) and *RL*_*2 chosen + unchosen*_ (*cyan*) as well as two control models (*RL*_*3*_ and *RL*_*4*_, *see* Materials and methods *for details*) and a Bayesian learner (BL). GLMs (*lower*) comparing explorative errors predicted by all five models (*RL*_*1–4*_ *and BL*) with those made by both monkeys during the last seven trials of a block. Analysis revealed *RL*_*2 chosen + unchosen*_ provided a significantly better prediction of explorative rules errors at the end of a rule block and a better overall prediction of the animals’ choices. Individual sessions indicated by single dots. Large circles denote the mean for each model. The data underlying this figure can be found via the following https://doi.org/10.12751/g-node.knk883.

To confirm whether this alternative strategy, requiring estimated values for both rules, could account for the monkeys’ behavior we modeled the value associated with each rule by fitting a number of RL models [24,25] to the choices made during each session (Figs 2 and S2). In the first model (RL_1 chosen_), only the value associated with the rule chosen by the animal was updated (on a trial-by-trial basis), with the value of the unchosen rule maintained until selected again. The second model (RL_2 chosen + unchosen_) differed in that the values associated with both rules were updated on every trial. That is both the value of the rule chosen by the animal, and the unchosen alternative were updated on every trial (note each rule was updated in the same direction, but with differing values for each learning rate, see Materials and methods for details). In addition, we fit two further RL models to exclude the possibility that these explorative errors arose because animals were increasingly unable to remember the correct rule to exploit throughout blocks, or because they were momentarily distracted and chose incorrectly (S2 Fig, RL_3 random noise_ and RL_4 point disruption,_ respectively), as well as a Bayesian learner (BL) [26].

Examination of the goodness-of-fit achieved by all four RL model variants, by means of a repeated measure ANOVA (Fig 2C, one between subject factor, Monkey, with two levels M1 and M2, and one within subject factor, Model, with five levels RL_1−4_ and the BL, see Materials and methods for details), revealed a significant difference between the five models (*F*_(4,64)_ = 267.1, *p* = 2.68 × 10^−32^). The fit achieved by updating the value of both the chosen and unchosen rules for each trial was significantly better than that from updating only the value of the chosen rule (Fig 1E, pseudo *r*^2^ for RL_2 unchosen + chosen_ versus RL_1 chosen_, 0.32 ± 0.009 versus 0.28 ± 0.01, respectively, Cohen’s *d* = 0.49, *p* = 6.3 × 10^−4^, posthoc *t* test with Bonferroni correction for multiple comparisons). By contrast, the fit achieved by including a random noise component (representing an inability to recall the correct rule), did not improve the model fit (pseudo *r*^2^ for RL_3 random noise_ 0.28 ± 0.081, Cohen’s *D*, 0.11 *p* = 0.61), while the inclusion of a function capturing single trial distractions within the model resulted in significantly worse fit than the original model (Pseudo *r*^2^ for RL_4 point disruption_, 0.26 ± 0.011, Cohen’s *D*, 0.25, *p* = 0.001). Finally, the pseudo *r*^2^ obtained for the BL indicated it fit the animals’ choices significantly worse than the standard RL model (pseudo *r*^2^ for BL 0.12 ± 0.07, Cohen’s *D*, 2.52 *p* = 1.01 × 10^−8^). Examination of model fit in both animals separately confirmed RL_2 chosen + unchosen_ provided a significantly improved goodness of fit in both animals (S3 Fig)

Session-by-session analysis of the explorative rule errors predicted by each RL model against the explorative errors made by the monkeys at the end of each block (Fig 2C) confirmed that the increase goodness of fit achieved by considering both the current and counterfactual rule (RL_2 chosen + unchosen)_ was due to improved prediction of explorative errors made by both animals. A further repeated measures ANOVA (one between subject factor, Monkey, with two levels M1 and M2, and one within subject factor, Model, with five levels RL_1−4,_ and BL see Materials and methods for further details) revealed that the prediction of explorative errors differed significantly depending on RL model (*F*_(4,64)_ = 4.3, *p* = 0.0036), and that the RL_2 chosen+unchosen_ provided a significantly better model of explorative rule errors made at the end of a block than the standard RL model (mean parameter estimate for RL_2 chosen+unchosen_ 0.30 ± 0.06 versus the mean perseverative error (P.E.) for RL_1_–0.05 ± 0.08 *d* = 1.05, *p* = 5.0 × 10^−4^), as well as both modified control models (mean P.E. for RL_3 random noise_, 0.07 ± 0.09, *d* = 0.73, *p* = 0.0019, and RL_4 point disruption_ 0.098 ± 0.07, *d* = 0.65, *p* = 0.013), and the BL (mean P.E. for BL −0.71 ± 0.05, *d* = 1.09, *p* = 4.0 × 10^−4^)

### Local field potential activity in FPC correlates with the counterfactual rule value and reward

**Fig 3:**
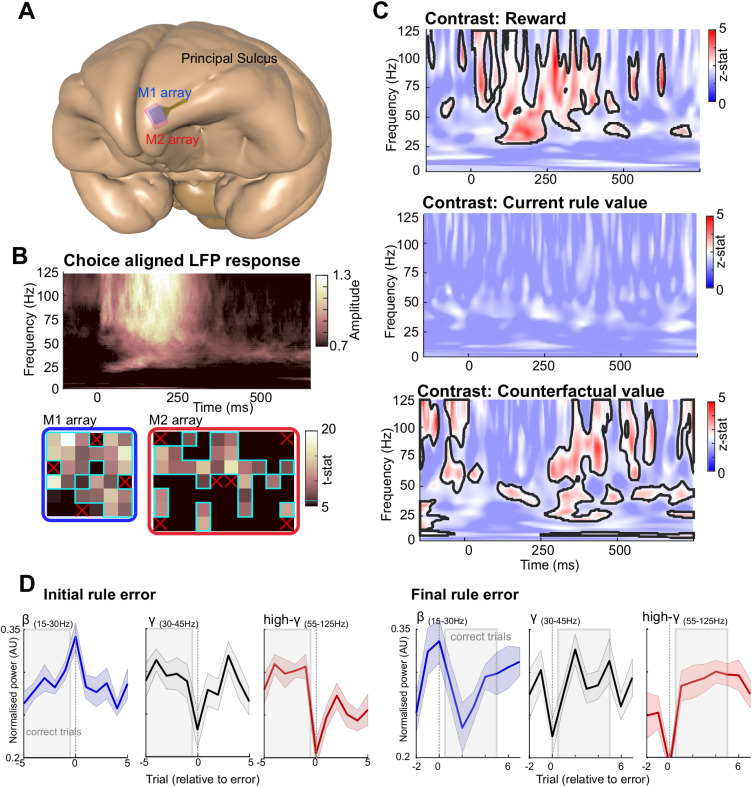
LFP activity recorded from FPC tracks the predicted value of the counterfactual rule. **A.** Illustration showing the location of Utah arrays implanted into FPC of M1 (32 channels, blue) and M2 (64 channels, red); poststudy analysis confirmed both arrays implanted in the most rostro-lateral portion of FPC, with the entire array located rostral to the anterior tip of Principal Sulcus. **B.** Choice-aligned spectrogram showing a strong gamma frequency response (*top*). Array maps showing choice responses across the arrays implanted in M1 (blue) and M2 (red). Electrodes with significant gamma responses aligned to choices outlined in cyan. Red crosses indicate reference electrodes **C.** Group level one-sample *t* test results from a GLM analysis. Significant clusters visible in choice-aligned results for reward and the value of the current and counterfactual rule (*top*, *middle*, and *bottom* panel, respectively, threshold *z* > 2.33 and cluster corrected at *p* < 0.05). The spectral content associated with reward delivery was primarily observed in the gamma frequency bands, while counterfactual rule was associated with bursts of gamma and beta activity.. **D.** Rule-error aligned, postchoice LFP activity in the beta (blue, 15–30 Hz), low gamma (gray 30–45 Hz), and gamma bands (red, 55–95 Hz). Activity shown relative to either an initial rule error (i.e., error preceded by a minimum of 5 correct trials; left two panels) or a final rule error (i.e., error followed by at least 5 correct trials; right two panels).

To determine the functional relevance of the LFP activity present in FPC (array locations shown in Figs 3A and S4) while monkeys performed the WCST, we first calculated spectrograms with alignments to key task-epoch onsets, namely on-screen presentation of the sample and the targets, as well as the moment monkeys made a choice to the screen which initiated feedback. These analyses revealed a clear and robust response in the high gamma frequency band (55–95 Hz) which peaked 200–220 ms after the animals expressed their choice (Fig 3B). Due to the short, fixed delay between the animals’ choices, and the reward delivery, it was not possible to dissociate whether this activity was due to the expectation of a reward or the reward itself. By contrast, there was limited evidence of gamma frequency activity aligned to the presentation of either the sample or targets (S5 Fig). Further analysis, calculated on an electrode-by-electrode basis, revealed that gamma activity aligned to the choice was widespread across the FPC-arrays in both animals (Fig 3B). The majority of electrodes in M1 (25 of 32 electrodes) and just under half in M2 (26 of 64 electrodes) exhibited significant gamma frequency responses without excessive 50 Hz mains noise (significant at *p* = 0.01, independent *t* test, not cluster corrected, see Materials and methods for details). Comparison of choice-aligned activity recorded from both monkeys revealed no significant difference in the peak frequencies observed in both animals (S6 Fig, repeated measure ANOVA Monkey by Frequency interaction, *f*_(2,26)_ = 3.31, *p* = 0.067).

Averaging of the activity observed in FPC during shape and color trials, revealed no significant difference in the LFP activity associated with the two abstract rules (S7 Fig). Therefore, we conducted a session-by-session GLM analysis to link the choice-aligned spectrogram with time-series encoding behavioraly relevant information. Given previous reports emphasizing the importance of transient changes in the LFP activity recorded from the prefrontal cortex [27], this GLM analysis compared fluctuations in LFP activity, on a trial-by-trial basis against the values obtained from the best-fitting RL_2 chosen+unchosen_ model. In line with the hypothesized role of FPC, this analysis included regressors relevant to both exploitative and explorative behavior, namely reward delivery, the predicted value of the current, and counterfactual rule, as well as the prediction error of both rules (S8 Fig). Current and counterfactual rule estimates were obtained by converting shape and color values obtained from the RL model. Current rules values consisted of the value of whichever rule was correct for each block, with the opposite true for counterfactual rule value (see Materials and methods for further details). Spectrograms displaying the *t*-stats obtained from the group level analysis revealed significant activity in both the low (30–45 Hz) and high gamma frequency (55–95 Hz) bands, associated with reward delivery. The onset of this activity coincided with animal’s choice and extended for a further 300 ms, corresponding to the period during which animals received rewards for correct choices (Contrast: Reward; peak *z*-stat: 4.92, 93 Hz and 268 ms, Fig 3C). In addition, these analyses revealed bursts of activity, occurring predominantly 400–600 ms after the animals made a choice which were significantly associated with the predicted value of the counterfactual rule. Although the peak of this activity was in the high gamma frequency band (Contrast: Counterfactual Value; peak *z*-stat postchoice: 3.92, 80 Hz and 433 ms), it extended to lower frequencies and included a strong beta frequency component. Analysis of the activity observed in both animals confirmed this observation. While gamma frequency activity (including both low and high gamma activity) was associated with both reward delivery and counterfactual value, beta frequency activity was only associated with counterfactual value (S9 Fig). Finally, there was no evidence that choice-aligned neuronal activity encoded the predicted value of the current rule (Fig 3C), nor the prediction errors obtained from the RL models which related to either the current or counterfactual rules (S8 Fig).

**Fig 4:**
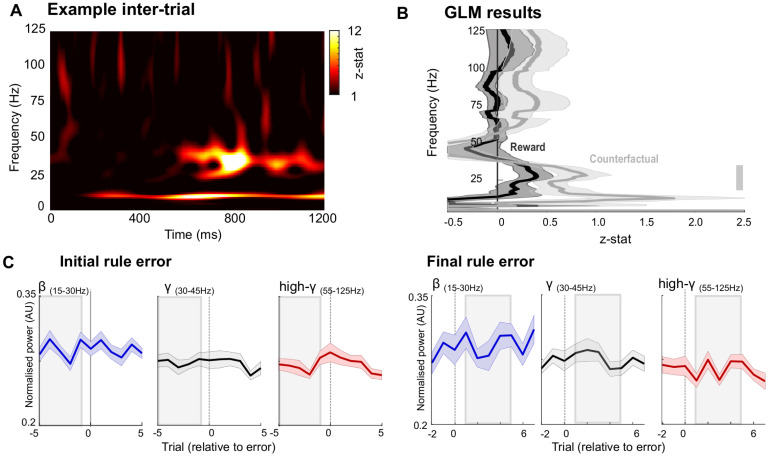
Inter-trial interval local field potential activity in FPC. **A.** Example spectrogram from a single period of 1.2 s of inter-trial interval activity from FPC, taken directly after the end of the trial (see Materials and methods). LFP activity in FPC dominated by low frequency activity (<50 Hz) **B.** Power spectra showing the results of two contrasts (counterfactual value, gray; reward, black) from a session-by-session GLM analysis (see Materials and methods). Significant association shown between counterfactual value and beta frequency activity shown by gray bars (*p* < 0.05, one-sample *t* test, cluster corrected at *p* < 0.05). **C.** Rule-error aligned, inter-trial LFP activity in the beta (blue, 15–30 Hz), low-gamma (gray, 30–45 Hz), and high-gamma bands (red, 55–95 Hz). Activity shown relative to either an initial rule error (i.e., error preceded by a minimum of 5 correct trials; left two panels) or a final rule error (i.e., error followed by at least 5 correct trials; right two panels).

To better understand the relationship between LFP activity recorded from FPC and the animals’ decisions, we performed two additional analyses. Firstly, we examined changes in the power of beta, low and high gamma frequency activity relative to rule errors made by both monkeys (Fig 3D). Secondly, we analyzed inter-trial activity in FPC to determine whether there was any relationship between persistent, background activity in FPC and counterfactual value (Fig 4).

The power of beta and gamma activity relative to either an initial rule error (i.e., a rule error preceded by at least 5 correct trials) or a final rule error (i.e., a rule error followed by at least 5 correct trials) was highly dissociable (Fig 3D). Beta activity in FPC peaked around the time of the feedback immediately following a rule error (i.e., on trial zero in Fig 3D). Furthermore, the magnitude of feedback-associated beta activity increased across runs of consecutive correct trials (observed both in the run up to an error shown in initial rule error plot, and after a rule error shown in final rule error plot). In stark contrast, the power of high gamma activity in FPC was lowest in the feedback epoch immediately following a rule error, and during runs of consecutively correct trials high gamma activity remained consistently high. A similar pattern was evident for low gamma activity, which was lowest for rule errors, and stronger for correct trials (although with greater variability than observed for high gamma activity (Fig 3D).

Taken together, these observations, that postchoice beta frequency activity in FPC follows the counterfactual value obtained from RL models (Fig 3C), and that peak power increases over consecutive correct trials (Fig 3D), suggest that beta frequency activity within FPC may coordinate long-term maintenance of value representations between decisions. To evaluate the generality of the timing of this relationship, we examined LFP activity recorded from FPC during the corresponding intertrial intervals (i.e., an epoch 1.2 s after the end of the trial, see Materials and methods, and Fig 4). The LFP recorded from FPC during the inter-trial interval was dominated by activity in lower frequencies (<50 Hz, Fig 4A). Consistent with the above results, linking choice-aligned beta activity with counterfactual value, GLM analyses of activity during this intertrial period revealed a relationship between beta frequency activity (15–30 Hz) and counterfactual value (peak *z*-stat 2.73, 22 Hz Fig 4B). However, analyses of changes in the amplitude of this activity relative to rule errors revealed no direct relationship between either beta, low, or high gamma frequency activity and the errors or correct choices made by the animals (initial rule error and final rule error plots; Fig 4C). These findings suggest that while the amplitude of offline (intertrial interval) beta activity in FPC does contain information relating to counterfactual value, it is of no direct relevance to the animals’ decisions (correct or otherwise). Importantly, this implies the within-trial-related activities (Fig 3C and 3D) are not sustained activities maintained across the inter-trial interval, rather they are dynamically reconfigured around the time of decisions.

### High-frequency microstimulation of FPC impairs adaptation to rule changes and perturbs local field potential activity in FPC

**Fig 5:**
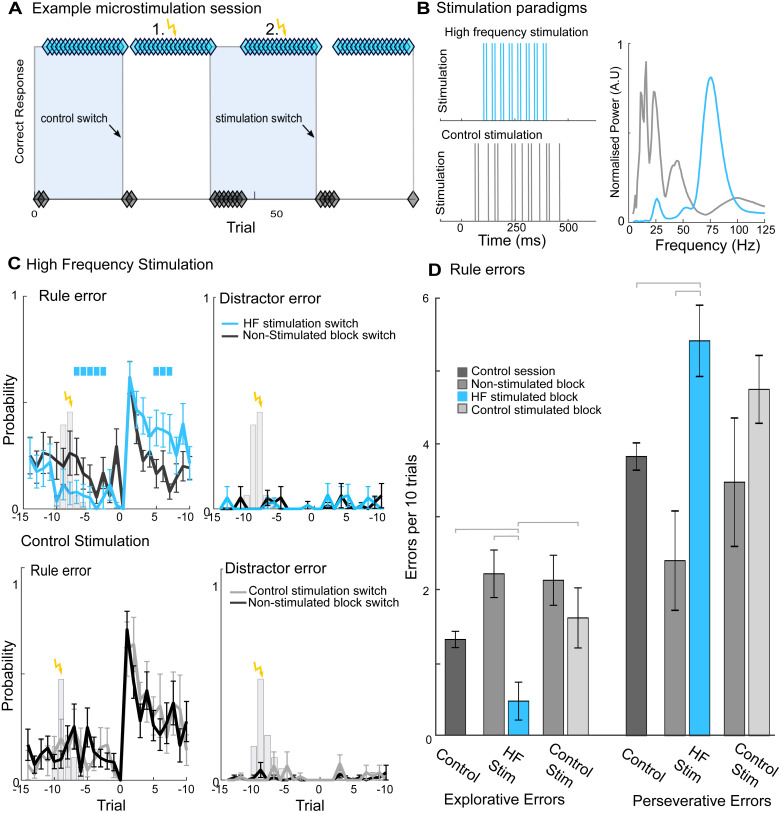
Modulation of monkeys’ behavioral performance by high-frequency electrical microstimulation of FPC. **A.** Illustration showing the stimulated trials within an example microstimulation and recording session. Microstimulation was triggered by the reward TTL pulse, after monkeys had achieved 85% correct over 10 trials (indicated in yellow). Microstimulation was delivered to a single trial in a pseudorandom selection of both color and shape blocks in a session. Control and poststimulation block changes indicated with black arrows. Diamonds denote correct (cyan) and incorrect trials (black). **B.** Illustration of the two microstimulation protocols, high-frequency (*blue)* and control stimulation (*gray, both shown on left*), and the LFP spectral content of both protocols recorded during stimulation from FPC (*right*). See Materials and methods for details of the stimulation protocols. **C.** Plots showing the number of rules errors and distractor errors made by monkeys at the block change following microstimulation. High-frequency (*blue, upper*) and control microstimulation (*gray, lower*) sessions compared to nonstimulated blocks from within the same behavioral session (*black*). All data shown mean of both monkeys ±SE. Significant differences between stimulation and nonstimulation indicated with color bar, and tested using unpaired t-tests (see Materials and methods). All *p*-values corrected using Bonferroni correction. Gray bars denote probability of single-trial stimulation occurring (relative to block change). **D**. Comparison of explorative and perseverative errors made following high-frequency (blue), and control stimulation as well as for nonstimulated block changes within both stimulation sessions and block changes during nonstimulation control sessions. Significant differences determined by Bonferroni corrected posthoc *t* tests. The data underlying this figure can be found via the following https://doi.org/10.12751/g-node.knk883.

The preceding data demonstrate the co-existence of two task parameters evident in the LFP recorded from FPC. While not completely dissociable, our data suggest gamma activity was associated with reward delivery (and a correct trial outcome), while beta frequency activity tracked the predicted value of the counterfactual rule. These correlational observations support the hypothesis outlined earlier that FPC is involved in redistributing resources towards alternative goals (i.e., to counterfactual rules in this case). To directly determine causal links between FPC and decision-making we next used electrical microstimulation via the implanted electrodes. Microstimulation at high frequencies has previously been observed to perturb both the physiological function of local circuits and emergent cognitive processes [28,29]. Based on these studies and our aforementioned observations of gamma activity within FPC we chose a “high frequency” stimulation protocol with a peak stimulation frequency of 75 Hz, consisting of 16 pulses, with alternating inter-pulse intervals of 13 ms (75 Hz) followed by 40 ms intervals (Fig 5A and 5B, and Materials and methods for more details). On each pulse 3 electrodes were stimulated in FPC from a wider selection of 6 electrodes, with the 3 chosen changing on each pulse in the train (see S10 Fig and Materials and methods). Microstimulation was delivered on a single trial within each “stimulated block” and was triggered by the reward delivery once animals attained >85% across 10 trials (hence typically occurring ~8–10 trials prior to the next rule reversal, see Fig 5 and Materials and methods). Blocks to be stimulated were chosen pseudorandomly, and approximately 40% of blocks within stimulation sessions had this single trial stimulated therein, with the rest remaining nonstimulated control blocks. Spectral analysis of the stimulation artifact generated within FPC confirmed that this stimulation caused two pronounced frequencies of activity in the LFP, one at 75 Hz and one at 25 Hz (Fig 5B).

We hypothesized that this artificial high-frequency stimulation of FPC should affect the region’s influence on cognition, either counterfactual valuation (mediated by bursts of gamma and beta activity) or reward processing (associated with gamma activity alone). Accordingly, these two hypotheses predict contrasting behavioral changes. The first hypothesis supposes disruption to the encoding of the predicted value of the counterfactual rule in FPC. According to this hypothesis, the disruptive stimulation would disrupt or suppress the representation of the value of the counterfactual rule, leading in turn to fewer explorative rule errors, and improved behavioral performance in trials that are poststimulation but prior to the upcoming rule/block change. As anticipatory counterfactual rule-value information is beneficial for rapid switching, this hypothesis also predicts increased perseverative errors at the first poststimulation rule switch reflecting the need to relearn the information wiped out (or suppressed) by microstimulation. The second hypothesis concerns disrupted reward representations in FPC. According to this hypothesis, the stimulation might be expected to impair reward-based rule consolidation before the upcoming rule/block switch, thereby increasing uncertainty-triggered exploration (i.e., more explorative rule errors) in the trials running up to the rule/block change. This second hypothesis also predicts impaired performance postrule/block change because it might be expected to impair reward-based rapid updating of relative rule values. In addition, we utilized a “control stimulation” protocol, delivered in separate behavioral sessions, in which the inter-pulse interval was a pseudo-random integer from 5–100 ms. This stimulation protocol was not expected to induce marked disruption to LFP activity within FPC; indeed, analysis of the stimulation artifact associated with this protocol confirmed the absence of high-frequency activity (activity extended from 15 to 45 Hz, Fig 5B).

Our initial analysis of stimulation induced changes in animals’ behavior examined changes following stimulation relative to nonstimulated blocks within the same session. Alignment of the animals’ behavior with the stimulated trial did not reveal an immediately discernible change in the animals’ behavior after either stimulation protocol (S11 Fig). However, as the animals approached the subsequent rule change marked changes were evident in the probability of monkeys making rule errors following the high-frequency stimulation protocol (Fig 5C).

During the intervening trials poststimulation but prior to the subsequent rule switch, monkeys made significantly fewer explorative errors following high-frequency stimulation than for nonstimulated blocks (probability of making explorative errors significantly lower from 3 to 7 trials prior to rule switch, *p* < 0.05 cluster corrected, the effect peaked three trials prior to rule switch prior to rule switch, 0.17 versus 0.05 in no stimulation and high-frequency stimulation, respectively, Cohen’s *d* 1.50, *p* = 0.004). In addition, there was a significantly higher probability of monkeys making perseverative rule errors in the trials immediately after the first poststimulation rule switch following high-frequency stimulation than during nonstimulated blocks (probability of making perseverative errors significantly lower than from trials 5 to 7 postrule switch, this effect peaked five trials postrule switch, 0.16 versus 0.38 in no stimulation and high-frequency stimulation, respectively, Cohen’s *d* 1.96, *p* = 0.04). By contrast, high-frequency stimulation had no effect on the probability of animals choosing the distractor target either before or after the poststimulation rule switch (probability of making distractor errors for all trials pre- and postrule switch was *p* > 0.05, Fig 5C).

Comparison of the monkeys’ performance following control stimulation protocol (without a high-frequency component, Fig 5B) with nonstimulated trials from the same session did not reveal any significant changes in the monkeys’ ability to perform the WCST. There was no significant difference between the probability of monkeys making rule errors with control stimulation versus no stimulation either prior to (probability of making a perseverative error peaked four trials before block change 0.15 in nonstimulation blocks versus 0.10 in control stimulation blocks, Cohen’s *d*, 0.58, *p* = 0.33) or following the block change (probability of making a perseverative error peaked five trials after the block change 0.20 in nonstimulation blocks versus 0.26 in control stimulation, Cohen’s *d*, 0.59, *p* = 0.30). Consistent with the previous results, control stimulation did not change the probability of animals making distractor errors either before or after the subsequent rules switch (Fig 5D).

Further analysis utilizing repeated measures ANOVAs to compare the number of explorative and perseverative errors made by both animals across behavioral sessions confirmed these trends (two within-subject factors: Stimulation protocol, 3 levels (HF stimulation, control stimulation, and no stimulation) and BlockType, 2 levels (stimulated and nonstimulated), see Materials and methods, Fig 5D). There was a significant main effect of stimulation protocol for both explorative errors and perseverative errors (*F*_(2,26)_ = 15.62, *p* = 8.18 × 10^−7^ and *F*_(2,26)_ = 14.17, *p* = 6.89 × 10^−5^, respectively). Furthermore, there was a significant interaction between stimulation protocol and block type for both explorative and perseverative errors (*F*_(4,52)_ = 21.18, *p* = 2.079 × 10^−10^ and *F*_(4,52)_ = 37.403, *p* = 1.014 × 10^−14^, respectively). Posthoc *t* tests confirmed that the number of explorative errors was significantly lower following high-frequency stimulation than after control stimulation (*p* = 0.0052, mean explorative errors 0.47 ± 0.26 versus 1.62 ± 0.41 per session for high-frequency and control stimulation, respectively) or when compared to nonstimulation sessions (*p* = 0.024, mean explorative errors 1.32 ± 0.11 per session for nonstimulated sessions).

By contrast, perseverative errors made after poststimulation block changes by both animals were significantly higher after high-frequency stimulation than at block changes during nonstimulated sessions (*p* = 0.035, mean perseverative errors 5.42 ± 0.50 versus 2.40 ± 0.68 for high-frequency stimulation and nonstimulated session, respectively). Although the animals made more perseverative errors following high frequency than following control stimulation, the difference was not significant (*p* = 0.24, mean perseverative errors 4.75 ± 0.47 following control stimulation). Finally, there was no difference between the number of explorative or perseverative errors made during nonstimulated blocks from either stimulation protocol or control nonstimulated session.

Taken together, the pattern of errors shown in these results (decreased explorative rule errors following stimulation up to rule-change, coupled with increased perseverative errors after the rule-change, and no evidence of increased distractor errors) is consistent with our first hypothesis that high-frequency stimulation disrupts FPC-mediated behavior based on its encoding of the predicted value of the counterfactual rule. Previous studies have suggested that negative correlations between explorative and perseverative errors would reflect a common exploratory drive [30]. Analysis of the relationship between both error types revealed a significant negative correlation between explorative and perseverative error types in nonstimulated sessions (*r* = −0.045, *p* = 1.80 × 10^−5^ S12 Fig). Following HF microstimulation there was no correlation between explorative and perseverative errors in stimulated blocks (*r* = −0.07, *p* = 0.68), although the negative correlations between errors were still evident in nonstimulated control blocks from the same session (*r* = 0.30, *p* = 0.018). By contrast following control stimulation explorative and perseverative errors remained significantly correlated in stimulated blocks (*r* = −0.32, *p* = 0.049), while there was a negative but nonsignificant correlation between explorative and perseverative errors in nonstimulated control blocks from the same sessions (*r* = −0.25, *p* = 0.07).

**Fig 6:**
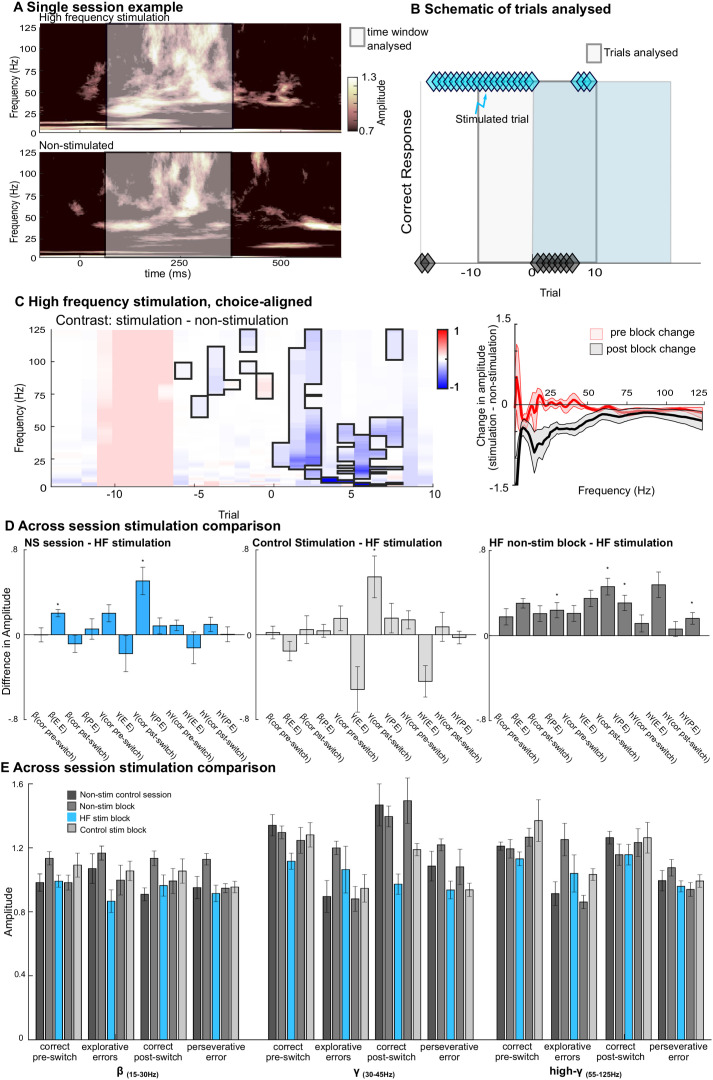
Disruption of choice-aligned LFP activity in FPC following high-frequency electrical microstimulation. **A.** Example spectrograms showing choice-aligned activity recorded from FPC following high-frequency stimulation (*upper*) or in the absence of stimulation (*lower*). Spectrogram calculated by averaging over the fifth to eighth trials after stimulation. Nonstimulation spectrogram calculated from the average spectrograms of trials from the last four trials of four nonstimulated blocks (i.e., same relative block epoch). White box indicates the 400 ms period used for further analysis. **B.** Illustration of the trials included in the analysis of changes in LFP activity recorded from FPC caused by electrical microstimulation. In stimulation blocks, the 10 trials before and 10 trials after the poststimulation block change were included (white box). In nonstimulation blocks a corresponding window of trials was selected. **C.** Trial-by-trial spectral analysis showing the difference between the field potential in FPC following high-frequency stimulation and nonstimulated blocks within the same session **D and E**. Cross session comparison showing **D.** the difference in amplitude between nonstimulated control sessions and high-frequency stimulation (*left*), control stimulation sessions and high-frequency stimulation (*middle*) and between nonstimulated blocks and high-frequency stimulated blocks (*right*) split for beta (β) (15–30 Hz), gamma (γ) (30–45 Hz) and high gamma (_h_γ) (55–95 Hz) bands and for explorative (E.E.) and perseverative (P.E.) errors as well as correct trials pre block switch (cor preswitch) and correct trials postblock switch (cor pst-switch). **E.** LFP amplitude for nonstimulation control sessions (dark gray), stimulated blocks from high-frequency (blue) and control stimulation (light gray), as well as nonstimulated blocks from both stimulated sessions (gray). Amplitudes are shown using the same split as used in **D.** The data underlying this figure can be found via the following https://doi.org/10.12751/g-node.knk883.

Finally, to determine if our electrical microstimulation protocols caused an enduring effect upon the LFP activity in FPC concomitant with the behavioral change (associated with both hypotheses), we compared changes in LFP activity recorded from FPC after both of the electrical microstimulation protocols (Fig 6). This analysis compared 400 ms of choice-aligned activity for 10 trials before and 10 trials after the first poststimulation block change (see Fig 6A and 6B and Materials and methods) with corresponding activity recorded before and after nonstimulated block changes from within the same session. A repeated measure ANOVA with one between-subjects factor: Monkey (two levels, M1 and M2) and one within-subjects factor: Stimulation (two levels, stimulation and nonstimulation) revealed significant differences between LFP activity recorded following high-frequency microstimulation and nonstimulated activity (Fig 6C). This analysis revealed two clusters which were significantly different between high-frequency stimulated and nonstimulated trials. Firstly, following high-frequency microstimulation activity in the high gamma activity (55–95 Hz, peak *z*-stat 2.81, 90 Hz, three trials preblock change) was significantly lower than in nonstimulated blocks prior to the subsequent rule-switch and associated block change (trial 0). In addition, a second cluster of decreased activity was evident postrule-switch. This cluster was evident over the first eight trials after the change in rule and was predominately in the low gamma frequency (*z*-stat 3.08, 24 Hz, one trial postrule switch) and beta frequency band (peak, *z*-stat 3.74, 44 Hz, eight trials postrule switch), although it extended into the high gamma frequency bands immediately after the rule switch (Fig 6C). By contrast a comparable analysis between control stimulation, and nonstimulation trials did not reveal any significant differences in field potential activity recorded from FPC, either before or after the block change (S13 Fig).

To confirm these changes in activity within FPC, we compared LFP recorded from FPC following both stimulation protocols within activity recorded from nonstimulated control sessions (Fig 6Dand 6E). Consistent with results presented in Fig 3, these data (Fig 6E) revealed strong gamma and high gamma responses and low beta frequency activity when the animals chose correctly; by contrast errors (both explorative and perseverative errors) were typified by both lower gamma and lower high gamma responses but increased beta frequency activity. Comparison of the difference in LFP amplitude between nonstimulation sessions and high-frequency stimulation sessions (Fig 6D left panel) revealed that the stimulation decreased the amplitude of activity in FPC for several trial types. Following high-frequency stimulation, but prior to the poststimulation block change beta activity recorded from FPC for explorative errors was significantly lower than corresponding trials recorded from nonstimulation sessions (difference between nonstimulation and high-frequency stimulation explorative errors 0.2 ± 0.03, *p* = 0.0042 one-sided *t* test, Holm–Bonferroni correction for multiple comparisons). In addition, low gamma amplitude for correct trials made poststimulation but preblock change was lower than for nonstimulation sessions, although not after correction for multiple comparisons (difference between nonstimulation and high-frequency stimulation correct trials preblock change 0.2 ± 0.08, *p* = 0.045 one-sided *t* test, uncorrected). Furthermore, gamma activity recorded during correct trials following a block change was significantly lower after high-frequency stimulation than for corresponding trials from nonstimulation sessions (difference between nonstimulation and high-frequency stimulation correct trials postblock change 0.51 ± 0.13, *p* = 0.004, Holm–Bonferroni correction for multiple comparisons). The amplitude of gamma activity recorded during correct trials postblock change was also significantly lower following high-frequency stimulation than following control stimulation (difference between control stimulation and high-frequency stimulation for correct trials postblock change 0.55 ± 0.2, *p* = 0.0045 one-sided *t* test, Holm–Bonferroni correction for multiple comparison, Fig 6D middle panel).

Comparison of LFP activity following high-frequency stimulation and nonstimulated blocks from within the same sessions (Fig 6D right panel) revealed a comparable decrease in gamma for correct poststimulation trials (difference between nonstimulated blocks and stimulated blocks from high-frequency stimulations sessions for correct preblock change trials 0.43 ± 0.7, *p* = 0.0001 one-sided *t* test, Holm–Bonferroni correction for multiple comparison), as well as a drop in the amplitude of LFP amplitude across all bands for perseverative errors (difference between nonstimulated blocks and stimulated blocks from high-frequency stimulations sessions for perseverative errors 0.24 ± 0.07 *p* = 0.045, 0.25 ± 0.07 *p* = 0.002, 0.13 ± 0.03 *p* = 0.001 for beta, gamma, and high-gamma, respectively. All one-sided *t* test Holm–Bonferroni correction for multiple comparisons).

## Discussion

It has been proposed that FPC’s contribution to decision-making in primates is to track the relative value of alternative options, modulate exploratory versus exploitative tendencies, and so manage competing goals [2]. Little is known about the neural mechanisms that underlie such cognitive processes. Here using a WCST analogue task we present evidence of an association between rhythmic activity in FPC and two key variables needed to guide exploratory rule-guided decision-making, namely counterfactual rule value and reward delivery. Furthermore, we demonstrate causally that temporary disruption of FPC (using targeted electrical microstimulation) affects both the explorative decision-making behavior of the animals and the concomitant LFP activity in FPC. Specifically, brief FPC stimulation initially made animals better at the WCST task primarily due to reduced explorative errors in the run-up to a rule-change. In addition, rule shifting after subsequent block changes was impaired relative to baseline nonstimulated sessions, and to nonstimulated blocks from within the same session although not when compared to perseverative errors from control stimulation sessions. Importantly, the behavioral effect of stimulating during a single trial could still be observed up to around 20 trials later, and was accompanied by persistent changes in the amplitude of low gamma and beta activity in FPC. Taken together these results not only provide confirmation that FPC is essential for guiding exploratory decision-making but yield mechanistic insights into how neuronal activity within FPC can support rule-guided decision-making behavior.

Both of these stimulation-induced behavioral changes were consistent with the proposed role of FPC in contributing to guiding exploratory decision-making. Firstly, the decreased explorative behavior prior to the rule-change supports correlative evidence from human neuroimaging which links activity in FPC with switching behavioral choices [6,31] and exploratory decision-making [32,33]. Secondly, the increased perseverative error-rate after the rule/block change is consistent with patients’ perseverance with previously relevant abstract rules, as well as a slow adaption to new information following damage to or disruption of human FPC [12,34]. Intriguingly our results build upon previous research which suggests that a negative relationship between explorative and perseverative errors may reflect a common, tonic exploratory drive [30]. Here we consistently observed negative correlations between these error types in nonstimulated sessions. Following HF stimulation of FPC there was no relationship between exploratory and perseverative errors, suggesting that the common exploratory drive in this task is mediated by FPC. Given our modeling results, which demonstrate improved prediction of animals’ exploratory choices when counterfactual values are accounted for in the model, one interpretation is that this common exploratory drive, mediated by FPC is the counterfactual value. Interestingly, this decoupling of exploratory and perseverative errors following HF microstimulation of FPC suggests that FPC is not the only area involved in rule switching. This may provide an explanation for the mixed changes in perseverative errors we observed following HF and control microstimulation. While perseverative errors following HF microstimulation were significantly higher than during baseline nonstimulated sessions, and no-stimulated blocks from within HF sessions, there was no difference between the perseverative errors made during control stimulation sessions.

In addition, we present evidence that at least two forms of dissociable information can be observed in the LFP recorded from FPC. Gamma activity (extending across both the low and high gamma band) was directly associated with external information about successful trial outcome, while beta activity tracked the subjective (internally generated) value associated with choosing the counterfactual rule. A similar dissociation, between incoming sensory information and internally generated working memory, has been reported in the oscillatory activity recorded from lateral prefrontal cortex (LPFC); in macaque LPFC, gamma activity is pronounced during encoding phases of working memory tasks and is associated with incoming sensory information [35]. Comparable recordings from human LPFC have also reported gamma activity when remembering lists, where such activity scales with the information load of the items to be retained [36,37]. By contrast, beta activity in LPFC peaks in the absence of sensory information (e.g., during delay periods of working memory [27,35,38] and has been proposed to mediate top-down, internal signals [39,40], for example, strong beta frequency coherence directed from frontal to parietal lobes is a feature of directed spatial attention [41].

Here, both animals succeeded in the WCST by periodically exploring throughout blocks even though this generated some errors, albeit with a rate of error which still allowed block changes, and which avoided either extending blocks or decreasing the reward received by the animals. These explorative errors were made with comparable reaction times to correct trials, suggesting that our animals planned the switch in rule well in advance of their choice, perhaps attempting to anticipate an upcoming block change. Our results suggest such advance planning is possible. For example, we demonstrate that encoding of the value of the counterfactual rule in FPC occurs during feedback from the previous trial, and together with knowledge of the previously chosen rule, as well as the cue for the current trial, animals could form decisions about which rule to choose well in advance of the choices being presented. However, we stress that our WCST analogue wasn’t designed to manipulate the point within a trial at which animals opt to switch rules, and this will need testing in future research. This strategy likely developed through the animals being sufficiently well-trained to possess both a firm concept of the two abstract rules themselves (demonstrated by both animals rarely making distractor errors), and an understanding that the current counterfactual rule will become valuable as more reward is gained from the current rule.

Our behavioral modeling, in which the standard RL model captured the animals’ adaptation to block changes but not their explorative errors made at the end of blocks, is consistent with the concept that both animals attempted to estimate or predict upcoming block changes. Here, we improved the prediction of explorative errors with a simple modification of the RL model such that the value of both rules (shape and color) updated on every trial (RL_2_). In this model, both values were updated in the same direction, albeit with the unchosen rule updated at a slower and independently chosen learning rate. While it is theoretically possible that other models would yield comparable results, we were unable to achieve improvements in the prediction of explorative errors with several alternative models (including both Bayesian- and RL-based). Furthermore, this simple change, linking the value of both rules, provided a means to achieve flexible behavior while still only learning from the outcome of one’s behavioral choice, and a single, clear account of the animals’ behavior.

Within this framework, the co-existence of both internally generated predictions and sensory information within FPC is particularly notable. Here after a correct choice we observed a consistent high-gamma frequency, and a strong, if noisy, low-gamma response, together with a variable beta frequency component which increased in amplitude across runs of successive correct trials and peaked after rule errors. Interestingly, high-frequency microstimulation did not selectively disrupt either the gamma or beta LFP component as we hypothesized. Rather, following microstimulation we observed decreased low-gamma (following correct trials) and beta activity (following explorative errors), raising the possibility that activity in both bands is linked during decision-making.

Previous studies have suggested that one feature of neuronal activity in macaque FPC is the evaluation of internally generated decisions [15,16] during task feedback. The combined nature of the gamma–beta response observed here could reflect the mechanism by which information regarding task success, relayed within the incoming gamma activity, is used to update the persistent counterfactual value in turn organized by beta frequency activity in FPC. Hence, a potential interpretation is that microstimulation impaired the updating of the counterfactual value within FPC leading to a degraded (or unreadable) counterfactual rule value, resulting in the animals performing better towards the end of blocks as current rule was not competing with counterfactual rule. By extension, this diminished counterfactual representation delayed adaptation to the subsequent rule change as the animals required longer to re-acquire and establish the value of the now relevant (previously counterfactual) rule. Importantly, while this choice-aligned activity in FPC was affected by microstimulation across several subsequent trials, persistent inter-trial activity was unaffected, suggesting that while the value associated with the alternative goal or option is updated within FPC following a decision, this information may then either be communicated immediately to other cortical areas needed for decision-making (and only later reconstituted in FPC) or else stored in FPC an activity silent manner analogous to that proposed for working memory [42]. However, there are several unanswered questions regarding the nature of the mechanism underlying the persistent changes in LFP activity and behavior we observed following microstimulation. The above interpretation is one possible explanation, but without improving our understanding of the firing of neurons within FPC relative to the values observed here, the effect of microstimulation on their firing and the nature and timing of their communication with other cortical areas, it will remain unclear why we observe persistent changes in behavior following such brief microstimulation.

The transient nature of behavioral information relayed by neuronal activity recorded from FPC which we observed here is consistent with the majority of previous neuronal recordings from FPC which have reported task-relevant information (needed to guide future decisions) in the firing of FPC neurons only during the delay/reward feedback [15,16]. By contrast, recent findings from an object-in-place task found neuronal firing in FPC peaked during the initial presentation of a novel scene, but that it was possible to decode the position of the animals chosen location during the delay and around the moment of choice [19]. One parsimonious explanation for differences between these findings is that FPC is primarily engaged when the relative value of counterfactuals choices is uncertain and requires tracking across trials. Hence, in associative learning tasks (including the object-in-place task) the relative value of correct versus incorrect (i.e., counterfactual) targets is unchanged after the initial fast-learning phase (during which representations of relative values of novel stimuli are being discovered and accordingly susceptible to significant updates). With subsequent repeated presentations the task becomes an exercise in strengthening object-place associations, without the need to monitor counterfactuals or engage FPC, therefore the presence or complete absence of FPC becomes irrelevant for task performance [7]. Therefore, one potential explanation for the object encoding reported by Nougaret and colleagues is that it reflects signal from more posterior areas, and it would be fascinating to see such results with additional recordings from dorsolateral and ventrolateral prefrontal cortex to better understand this result.

There are notable structural and functional differences between the FPC of humans and macaques. In humans it has been proposed that a distinction exists between the medial, and lateral sub-division of FPC [2,5]. With the lateral sub-division reliably activated during tasks requiring directed exploration (e.g., cognitive branching [5,6,11]). By contrast, the medial sub-division is thought to mediate undirected exploration by allowing assessment of the value of current choices or behaviors. Importantly, it has been suggested that the lateral sub-division FPC, and by extension associated cognitive functions (such as cognitive branching) is a feature uniquely associated with humans, with macaque FPC thought to be analogous to the medial sub-division.

However, our results suggest the relationship between human and macaque FPC may be more complex than previously considered. Here we recorded from the most anterior-lateral region of macaque FPC and found activity related to both the value of the counterfactual rule as well postchoice feedback. Furthermore, our animals’ exploration of the counterfactual rule while rarely making distractor errors, appears to be a form of directed exploration which was disrupted by microstimulation of FPC. One interpretation is that macaque FPC is not simply homologous to human medial FPC alone, rather it may support some combined functions associated with both medial and lateral FPC in humans. By extension, the greater cognitive complexity evident in human explorative behavior may not merely reflect a network with the addition of a single unique cortical area in humans but instead relate to more nuanced differences between humans and macaques. For example, we note not only expansion of FPC as a proportion of brain volume noted in humans [43], but also the coexistence of independent processing streams within FPC [2,5], as well as the innervation of a broader network of regions through the connections of the lateral subdivision [44]. However, understanding of the functional homologies between human and macaque frontal pole remains nascent, and there are several important considerations. Firstly, lateral FPC is reliably activated in humans during complex behavior such as cognitive branching [5,6]. Cognitive branching requires a more structured consideration of counterfactual task rules than the WCST analogue studied here (i.e., retaining knowledge of which counterfactual rule to return to) and it is not clear whether macaques possess the cognitive ability to perform this highest level of directed exploration. In addition, the role of FPC within wider cortical networks is an ongoing area of research, and the functional implications of the distinct cortical network connected to lateral FPC remains to be determined. Future work may aim to selectively inactivate subdivisions of FPC to determine to what extent a medial/lateral functional subdivision is evident in macaque FPC across a wider range of behavioral tasks.

Dysfunction of FPC is common in patients with obsessive compulsive disorder, where under-recruitment of this lateral subdivision into the cortical networks supporting decision-making has been associated with deficits in set-shifting [45], task load [46], and emotional decision-making [47]. By contrast, FPC hyperactivity is observed in ADHD [48]. Indeed, significant developmental changes in FPC are associated with disturbed behavior in both ADHD [49] and Schizophrenia [50]. The nature of neuronal activity within human lateral FPC remains to be determined, however, our results suggest that coordinated neuronal activity at beta frequency may be crucial to normal functioning of FPC. Future research characterizing the dynamics between FPC and the wider network of cortical areas with which it is connected [51,52], will be essential to understanding how maladaptive cortical networks involving FPC can influence decision-making as well as targeting interventions to ameliorate sub-optimal decision-making.

## Materials and methods

### Animals

We recorded the LFP from, and performed electrical microstimulation of, FPC in two male macaque monkeys (*Macaca mulatta*) while they performed the WCST analogue [22]. At the time of recording, monkeys were aged 8 and 9 years, and weighed between 10 and 12 kg. Both monkeys were socially housed in enriched environments with a 12-hour light/dark cycle and had *ad libitum* water access. All animal surgery, anesthesia, and experimental procedures were carried out in accordance with the guidelines of the UK Animals (Scientific Procedures) Act of 1986, licensed by the UK Home Office, and approved by Oxford’s Committee on Animal Care and Ethical Review (PP5227475).

### Apparatus

The WCST analogue was programmed using Turbo Pascal (Borland), run under DOS on a desktop PC. The animal was sitting in a primate chair (Rogue Research) in front of the touch screen with the head-fixated and performed the task in a magnetic-shielded box. A window in the front of the chair provided its access to the touch-screen and a touch-sensitive knob which we refer to as a “key-touch” device that the animal had to steadily hold at various times in each trial and which was positioned low down in front of the touch screen. A peristaltic pump device located on top of the box fed trial-by-trial smoothie reward through a tube and to a spout positioned in the vicinity of the animal’s mouth. Below the screen was also an automated lunch-box which contained the majority of the animal’s daily meal (wet mash and fruits and nuts, etc.) and which only opened immediately at the end of the task thereby providing an additional and main motivation to the NHP to complete the session sooner rather than later (as can be achieved by good and focused task performance).

### Behavioral task

Prior to training on the WCST analogue both monkeys underwent preliminary training and shaping, consistent with procedures described previously [22,53]. Both monkeys were then trained to perform the WCST macaque analogue as previously described in detail (see [9,22] for full task-training stage-by-stage details). The WCST task used in this study used 36 different stimuli comprising six colors (red, yellow, green, cyan, blue, and magenta) and six shapes (square, triangle, circle, hexagon, cross, and ellipse). The sample in each trial was selected at random (without replacement until the entire set had been used) from the 36 stimuli. In each trial, the test items were also selected from the same set of 36 stimuli, and at random (with the restrictions imposed by the necessity to generate either a low- or high-conflict trial). The locations of the three test items (i.e., to the left/right/bottom of the sample) were also chosen at random. Together, these stimulus selection procedures ensured that to succeed at the task the animals could not learn about rewarded objects, object-features, or object-location; rather they had to learn abstract matching rules (see below).

Briefly, on each trial of the WCST analogue, monkeys were presented with a white fixation cue, at which point they required to hold the key touch. They were then presented with one central sample stimulus, and following a delay of 1.5 s three peripheral choice stimuli. One of the choice stimuli matched only according to color of the sample, and another matched only according to shape of the sample, while the third-choice stimulus shared no features. Following the disappearance of the fixation cue, animals then made a choice using the touch screen. On correct trials reward was delivered following a further 200 ms. Reward delivery was deterministic (each correct trial was rewarded at a fixed volume and probability with incorrect trials unrewarded), rather than probabilistic as in other example of reversal tasks [54]. On a block-by-block basis the correct choice was determined by either color or shape. The monkeys received no cue about the currently reinforced rule and so had to determine the correct rule based on feedback from preceding trials. The rule switched periodically but only after monkeys achieved performance ≥85% correct in 20 consecutive trials. Prior to electrode implantation surgery both monkeys received extensive training on the WCST analogue, with additional training postimplantation. For recording only sessions of this study (collected at the beginning of the study) the animals completed an average of 5 blocks, (i.e., rule reversals) per recording session (6.25 for M1 and 3.5 for M2).

### Neuronal recording and electrical microstimulation

In addition to a titanium mount for head restraint (Gray Matter Research, Bozeman, MT) each monkey was implanted with a total of four or five Utah arrays (Blackrock Microsystems, Salt Lake City, UT) with platinum iridium-tipped electrodes. These arrays were connected to two 128-channel pedestals (256 channels in total) and arrays were implanted into FPC and orbitofrontal cortex, and dorsal and ventral lateral prefrontal cortex (dlPFC and vlPFC); M1 received an array implanted in ACC. The location of the arrays implanted into FPC were confirmed by examination of the brain postmortem and are shown in S4 Fig. At the time of this microsimulation study (16 months after implantation in M1 and 11 months after implantation in M2) neuronal activity was only consistently observed in both animals in the FPC arrays (a 32 channel Utah array in M1 and a 64 channel Utah array in M2). Neuronal data was collected using a 256-channel Cerebus System and concurrent electrical microstimulation was carried out using a CereStim 96 system (all Blackrock Microsystems Salt Lake City, UT).

The raw wideband LFP was sampled at 30 kHz and saved offline for further preprocessing. These data were then downsampled to 1 kHz and bandpass filtered between 4 and 150 Hz. Mains noise was removed by notch filtering using an equiripple FIR filter with an attenuation of 30 dB between-frequencies from 48 to 52 Hz, surrounded by a ± 1 Hz transition band. Any residual harmonics were removed using comparable filters at 100 and 150 Hz. After removal of mains noise data from all electrodes were visual inspected to ensure signal quality. Electrodes with suspect signal quality (6 electrodes from M1 and 34 electrodes from M2) were not included in further analysis. Data presented in this study were collected from 18 recording-only sessions (monkey 1: 8 sessions, monkey 2: 10 sessions) and 35 recording and stimulation sessions (monkey 1: 18 sessions, monkey 2: 17 sessions).

Electrical microstimulation consisted of trains of 16 or 14 100 μA, 200 μs biphasic pulses (cathodal first). Two different stimulation protocols were delivered to examine FPC function. During the high-frequency (HF-stimulation) protocol, the inter-pulse-interval alternated between 13 and 40 ms, this protocol induced a pronounced 75 Hz response in the LFP recorded from FPC (Fig 4). In the control stimulation protocol, the inter-pulse-interval was a pseudo-random integer from 5 to 100 ms. This stimulation protocol caused a strong low, but not high-frequency response, in the LFP recorded from FPC (Fig 4). HF stimulation and control stimulation were delivered in separate behavioral sessions. On each pulse, 3 electrodes were stimulated, the exact combination of electrodes varied on each pulse in the train between 6 electrodes preselected with strong gamma frequency activity aligned to reward delivery (Electrode locations and schematic showing electrode stimulation shown in S10 Fig). Electrode groupings were kept consistent across both stimulation protocols. In microstimulation sessions, the stimulation was delivered approximately half the blocks in the session, the blocks stimulated were selected pseudo randomly as the start of each session. Microstimulation was never delivered on the first block of the session. Overall microstimulation was delivered on approximately 40% of blocks in each session (stimulation delivered on average on 3.1 and 1.3 blocks out of 8.1 and 3.3 total blocks per session for M1 and M2, respectively). Stimulation was delivered on a correct trial once monkeys had achieved 85% correct over 10 trials and was triggered by the reward delivery.

### Data analysis

All data analysis and preprocessing was carried out using freely available toolboxes and bespoke scripts written for MATLAB 2018B (The MathWorks, Natick, USA). Data was imported to MATLAB using the NPMK toolbox (4.4.0.0), and spectral analysis of LFP was carried out using the FieldTrip toolbox [55]. The scalable brain atlas was used to visualize the macaque brain [56].

### Behavioral modeling and analysis

To aid our initial analysis of the animal’s behavior, we classified the monkeys’ incorrect responses into several error categories. When animals chose the stimulus corresponding to the incorrect/counterfactual rule these trials were deemed “rule errors”. We further use the term perseverative and explorative error to refer to rule errors made during the start of the block (when animals perseverated with the previously correct rule) and at the end of the block (when animals continued to explore the incorrect rule), respectively. For the purpose of the RT analysis in S1 Fig and the comparison of model-predicted explorative errors, these errors were deemed to be rule errors made in the final 7 trials of the block.

To determine the cause of the explorative errors made by monkeys while performing the WCST analogue the value attached to both matching rules (color and shape) was modeled with four variants of a RL model [24,25]. The first two models (RL_1 chosen_ and RL_2 chosen + unchosen)_ were included to test the hypothesis that the monkeys tracked both the current and counterfactual rule. In addition, two models were fit to test alternative hypotheses for the generation of explorative errors. Firstly, that explorative errors occurred because the animals had difficulty recalling the correct rule towards the end of blocks (RL_3 random noise_). Secondly that these errors simply reflect the animals becoming distracted on individual trials (RL_4 point disruption_).

In the first model (Fig 1 D, RL_1 chosen_), only the value of the rule corresponding to the chosen target (and not the unchosen) was updated. In this model, if on trial *n* the monkey chose shape then the value *vC* of the color rule was unchanged while the value *vS* of the shape rule for trial *n + 1* was given by.

*Vs*_(*n*+1)_ = *vS*(*n*) + *α* × *β*(*n*),

where the constant *α* was the learning rate, and *β*(*n*), the prediction error, was given by.

*β*(*n*) = *r*(*n*) − *vS*(*n*),

where *r*(*n*) was the reward obtained for trial *n.* The same procedure was followed when monkeys chose color to calculate *vC*, the value associated with color. Both values were left unchanged on trials in which the animals chose the other rule. For example, on a trial in which the monkey chose to match to shape, only the value *vS* will be increased, with *vC* unchanged as it wasn’t chosen. On the rare occasions that the animals chose the distractor (the target which matched neither shape nor color), neither value was updated.

In the second model (Fig 2D, RL_2 chosen + unchosen_), the value of both the chosen and unchosen rules were updated according to the above equations on every trial regardless of which rule was chosen. That is, on an example trial in which the monkey correctly chose to match to shape, both *vS* and *vC* will be updated, with *vS* and *vC* increasing to reflect the outcome of the trial. Note that the equations used to calculate the chosen (here *vS*) and unchosen (*vC*) were independent, and that two separate learning rates, *α*, were utilized, with the learning rate assigned to the counterfactual rule limited to a range of 0.005 to 0.5.

For the third model (S2A Fig, RL_3 random noise_), only the value of the chosen rule was updated on each trial, the value of the unchosen rule was left unchanged (as above for RL_1 chosen_). To represent the failure of the animals to assign value to the correct rule as blocks progressed, the updated value (*vS* or *vC*) was modified by a randomly generated decimal, which increased over the length of the block.

*Vs*_(*n*+1)_ = *vS*(*n*) + *α* × *β*(*n*) − *pr*,

where *pr* was a randomly generated value from 0 to 1, which increased with block length, normalized so that 1 was equal to the length of the longest block in each session (see S2 Fig for an example session of the random value).

The final RL model (RL_4 Point distraction_) was designed to capture the hypothesis that explorative errors arise from the monkeys incorrectly allocating value to the wrong rule for a single trial within the task (e.g., becoming distracted for a single trial). Similar to RL_1 chosen_ this model updated only the value of the chosen rule on each trial. However, the value of the chosen rule was switched with the unchosen value for single trials selected pseudo-randomly. Point Distraction trials were selected when an accumulation function, which increased from 0 to 1 during the block, crossed a stochastic threshold, which varied in turn between 0.75 and 0.95 (an example of the threshold and accumulation function shown in S2B Fig).

For all four models, the probability of choosing either rule was then estimated from the two values (*vS* and *vC*) using a softmax function. For example, the probability of choosing shape (*pS*) on trial *n* was given by.


$$pS(n)\;\;\;\;=\;\;\;\;\frac{exp\;(\tau \;\times \;vS(n)}{exp\;(\;\tau \;\times \;vS(n))\;+\;exp\;(\;\tau \;\times \;vC(n))\;}$$

where the constant *τ*, inverse temperature, determines the randomness of the decision.

### Model optimization and comparison

The optimum values for the constants *α* and *τ* were obtained by finding the combination of parameters which minimized the negative log likelihood of the predicted choices for each model.

To determine the model which best fits the monkeys’ actual choices, the Akaike Information Criterion (AIC) was calculated from the minimized negative log likelihood obtained for the four models for session (for reference the AIC values for all four models, and AIC_null_ for all sessions are shown in S3 Fig). Goodness of fit was calculated using Mcfaddens Pseudo-*r*^2^ [57,58] a metric summarizing goodness of fit, derived from the AIC for which models with pseudo-*r*^2^ values between 0.2 and 0.4 are considered to fit the observed data well [57].


$$\text{Pseudo}-r^{\text{2}}\;=\;\;\;\;\frac{\text{1}-{\text{AIC}}_{\text{model}}}{{\text{AIC}}_{\text{null}}},$$

where AIC_null_ was the AIC calculated from a null hypothesis that monkeys chose randomly between the three available choices (match shape, match choice, and distractor) on each trial.

A repeated measure ANOVA was calculated to assess differences in model fit between the four RL models and an additional BL. This ANOVA had one between-level factor, Monkey (two levels; m1 and m2), and one within-level factor, Model (five levels; RL model_1–4_ and BL). Posthoc *t* tests, corrected for multiple comparisons using Bonferroni correction, were used to assess differences in the model fit overall and for each monkey separately (data split by monkey are shown in S3B Fig).

Finally, session by session GLMs were used to confirm that improvements in fit in the RL model were due to better prediction of the explorative errors made during the final trials of a block. For each session, a single time series was created, corresponding to the observed probability of explorative errors being made during the 7 trials preceding a block change. A GLM then used to compare this time-series with the fit of predictor time series derived from the corresponding sections of predicted perseverative errors from all four RL-models (RL_1–4_) and the BL. The GLM results from individual sessions were combined using a further, second-level, repeated measure ANOVA with had one between-level factor, Monkey (two levels; m1 and m2), and one within-subject factor, Model (five levels; RL model_1–4,_ and BL).

Within-session differences in the behavioral performance of M1 and M2 following electrical microstimulation were assessed by examining the perseverative errors made by both monkeys, aligned to the subsequent block change. Unpaired *t* tests were used to assess the probability of monkeys making perseverative errors in stimulated and nonstimulated trials for the 7 trials before and after a block change. Corrections for multiple comparisons were made using Holm–Bonferroni.

Between-session differences in behavioral performance were examined across nonstimulation session, high-frequency stimulation sessions, and control stimulation sessions. Data from stimulation sessions were split into stimulated block changes and nonstimulated block changes, respectively, giving five conditions (nonstimulation sessions, high-frequency stimulation sessions stimulated block changes, high-frequency stimulation sessions nonstimulated block changes, control stimulation stimulated block changes, control stimulation nonstimulated block changes) The number of explorative errors (rule errors made over the last 10 trials of a block) and perseverative errors (rule errors made over the first 10 trials of a block) were averaged per session and differences between conditions assessed using two repeated measure ANOVA’s, for explorative and perseverative errors, respectively. These ANOVA’s had one between-subjects factor, Monkey (two levels; m1 and m2) and two within-subject factors, stimulation type (two levels; stimulated and nonstimulated), and stimulation protocol (three levels; control, high-frequency stimulation, and control stimulation). Posthoc *t* tests were carried out using Holm–Bonferroni correction for multiple comparisons.

### Analysis of the local field potential

To link the LFP activity recorded from FPC with behavioral cues, we conducted spectral analysis of the activity recorded from FPC. The first step in this analysis was to examine cue-aligned spectrograms for every electrode in the arrays implanted in FPC. For each electrode, we selected 1,000 ms (−400 ms to +600 ms) segments of LFP, aligned to one of three behavioral cues (sample onset, target onset, and choice).

Spectral analysis was carried out on a trial-by-trial basis to preserve induced neuronal activity (i.e., activity occurring with a variable latency after the relevant behavioral cue). Spectrograms were calculated for every trial from the aligned LFP segments using Morlet wavelets with frequencies ranging from 4 to 124 Hz in 1 Hz steps. The full-width at half-maximum (FWHM) ranged from 656 to 123 ms with increasing wavelet peak frequency. In the frequency domain, this corresponded to a FWHM from 1.5 to 45.5 Hz (See S14 Fig, [59]). Spectral decomposition was carried out using the FieldTrip toolbox (ft_specest_wavelet function, [55]). Induced single-trial spectrograms were then normalized to the mean spectral content of 800 ms of intertrial interval activity prior to the start of each trial (averaged both across all trials, and across the 800ms of activity) before further analysis.

After the initial spectral decomposition, a secondary analysis was conducted to identify electrodes with increased activity associated with either of the three behavioral cues. In this analysis, responsive electrodes were identified using paired *t* tests to compare the mean activity between 20 and 100 Hz, −400 to −200 ms precue with activity in the corresponding frequency bands 200–400ms after the cue.

A session-by-session univariate GLM was used to link reward-aligned spectral activity to regressors representing physiologically relevant variables on a trial-by-trial basis. This GLM included a regressor denoting whether monkeys received a reward on each trial as well as four regressors derived from the RL model_2 chosen + unchosen_ described above. Two of these regressors denoted the estimated value of the current correct rule (a single timeseries of the value of the correct rule, e.g., color in color block, across all blocks in a session) and the counterfactual rule (a single timeseries of the value of the incorrect rule, e.g., the value of the shape rule in a color block, across all blocks in session). Finally, two regressors were included which corresponded to the trial-by-trial difference in the two estimated values (delta of the current and counterfactual rules). These time series were rectified to remove negative values and convolved as above. To improve signal-to-noise all five regressors were convolved with a Gaussian function with a width of 4 trials.

The GLM for each time frequency point was given by:


$$\text{LFP}(t,f)\;=\;\beta \text{0}\;+\;\text{Rwrd}\;+\;{\text{Cur}}_{\text{Val}}\;+{\text{Alt}}_{\text{Val}}\;+\;\Delta \;+\;\Delta \;+\Sigma$$

where LFP(*t*,*f*) is a variable containing the amplitude of the reward-aligned spectrogram for a given time, frequency point across all trials in session. Rwrd is the animals rewarded trial outcomes, Cur_Val_ and Alt_Val_ are regressors for the trial-by-trial estimates of the value of the current and counterfactual rule as described above and Δ_Cur_ + Δ_Alt_ regressors corresponding to the un-signed change in the value of each rule as described above.

This GLM was applied to all time, frequency points in the reward-aligned spectrograms for each electrode separately, before averaging across electrodes to yield five parameter estimate (PE) spectrograms for each session. A one-sample *t* test was applied to each time-frequency bin of each PE of the five PE conditions to test the hypothesis that activity in each bin was associated with each regressor. The resulting five statistical summary spectrograms (one for each regressor) were then thresholded at *z* = 2.33 and cluster corrected at *p* < 0.05.

LFP activity during the intertrial interval (absent of task-relevant cues or external events) was analyzed using a comparable pipeline. For all electrodes included in the choice-aligned analysis (selected above) a 1.2 s data epoch a selected directly following the end of each trial. Wavelet decomposition was carried out as described above (including normalization to the mean spectra of the intertrial-interval). Session-by-session GLM analysis, using the same behavioral regressors, was carried out on the resulting normalized spectrograms. However, to account the highly variable nature of intertrial activity, without external events to align activity across trials. This intertrial GLM analysis was only carried out in the frequency domain. Values of the time by frequency spectrograms less than 1 (i.e., low than the session mean intertrial values) were removed from the before averaging across all 1.2 s of data in the temporal domain. The GLM was then applied to each frequency bin of the resulting mean power spectrum. As above statistical significance was determined using one-sample *t* tests, comparing the parameter estimates from all sessions for a frequency bin against a null hypothesis that there was no relationship with each regressor. The resulting five statistical summary power spectra (one for each regressor) were then thresholded at *z* = 1.66 and cluster corrected at *p* < 0.05 (again with 1,000 random permutations).

### Analysis of changes in LFP activity following electrical microstimulation

To determine whether electrical microstimulation had an impact on a particular frequency band of field potential activity recorded from FPC, we examined choice-aligned LFP activity, before and after the first poststimulation block change and compared this to corresponding activity recorded from block changes which were not preceded by microstimulation (Fig 5A and 5B). We calculated changes in the LFP caused by electrical microstimulation by averaging 400 ms of choice-aligned spectrograms from 20 trials aligned to either the first poststimulation block change (−10 to +10 trials) or a corresponding block change without a preceding microstimulation (nonstimulation).

To assess within-session changes in LFP amplitude, this procedure was repeated for all block changes (stimulation or nonstimulation) and two frequency by trial matrices were obtained per session by averaging across all stimulated and nonstimulated block changes, respectively. To test for statistical differences between activity during stimulated and nonstimulated block changes, the stimulation matrix was contrasted against the nonstimulated matrix to generate a difference matrix for each session. A one-sample *t* test was applied to each time–frequency bin of this group difference matrix to test the hypothesis that there was no difference between the stimulation and nonstimulation conditions. The resulting statistical spectra were thresholded at *z* = 1.66 and cluster corrected at *p* = 0.05 with 1,000 permutations used for the cluster correction). This procedure was repeated separately for both the high-frequency microstimulation and control microstimulation protocols.

A further analysis assessing changes in LFP amplitude across sessions was carried out on all three session types (control nonstimulation sessions, high-frequency stimulation sessions, and control stimulation sessions), as with the across-session behavioral analysis (see above) LFP amplitudes from the stimulation sessions were split into stimulated and nonstimulated blocks yielding five conditions. In addition, LFP amplitudes were split by trial type into four groups, explorative trials (rule errors in the final 10 trials of a block) and preblock change correct trials, perseverative errors (rule error in the final 10 trials of a block) and postblock change correct trials. Differences between trial types in each of the five stimulation conditions were assessed using one-sample *t* tests on the difference between the groups (e.g., the difference between beta amplitude in nonstimulation control sessions and stimulated blocks in high-frequency stimulated sessions). All *p*-values were corrected for multiple comparisons using Holm–Bonferroni correction.

## Supporting information

S1 Fig Reaction time for WCST trial outcomes. Bar charts showing the reaction times for monkey 1 and 2 for correct (gray) trials, as well as the three possible error outcomes. Rule errors (where the animals chose the incorrect rule) we split into two categories “perseverative errors” made by the animals over the first 7 trials of the block (blue) and “exploratory errors” made over the final 7 trials of the block (cyan). Distractor errors were made when the animals chose the target which didn’t correspond to either rule shown. (blue gray). Significant differences between the mean reaction times were tested with posthoc Bonferroni corrected *t* tests. The data underlying this figure can be found via the following https://doi.org/10.12751/g-node.knk883.
(PDF)

S2 Fig Additional noise-driven RL models. **A.** RL_3 Random noise_, values for shape and color predicted by RL_3_ were modified by addition of random noise to reflect decreased ability to remember the correct rule. Random noise increased in strength during the block. Example of the values of color predicted by RL_3_ (blue) after modification with randomly generated noise (*left*) with values obtained by RL1 shown for comparison (*black*). Correct trials denoted by light gray diamonds. Examples of the random noise component shown for 80 trials and all blocks of a single session (*right*). Mean random noise value ±SEM denoted with black and gray, respectively. **B.** RL_4 point disruption_, values for shape and color switched for a single trial to reflect animals momentarily assigning value on a single trial. Example of the values for color obtained from a single session by RL_3_ shown in blue with values for RL_1_ in black (*left*). The probability of the values assigned to color and shape switching increased after a block change until crossing a pseudo-random threshold (*right*, see Materials and methods). The upper and lower bounds of the threshold, and the accumulation function are shown in gray and black, respectively. Example of the pseudo-random threshold (black) and accumulation function (gray) shown for 80 trials.
(PDF)

S3 Fig Additional RL model fit. **A.** Akaike Information Criterium (AIC) used to calculate Mcfadden’s pseudo *r*^2^ shown for all four RL models against the null hypothesis, that animals chose randomly between the stimuli on each trial. **B.** Analysis of the pseudo *r*^2^ values obtained for all four RL models shown for both M^1^ and M^2^ separately. RL_2 chosen + unchosen_ (light blue) provided a significantly better fit than the other three models in both animals. Asterisk denotes significant differences between model fit at *p* < 0.05). The data underlying this figure can be found via the following https://doi.org/10.12751/g-node.knk883.
(PDF)

S4 Fig Electrode array locations in M1 and M2. **A.** Diagrams showing the location of FPC arrays in M1 and M2 confirmed by postmortem examination of both animals’ brains. Note the difference in array size (32-channel and 64-channel arrays for M1 and M2, respectively).
(PDF)

S5 Fig Field potential activity in FPC is not modulated by onset of cue or target stimuli. Mean spectrograms calculated from electrodes in FPC showing the absence of gamma activity (*left panel*) and maps showing the limited response in gamma activity observed in arrays implanted in M1 and M2 (*right panels*) aligned to **A.** Cue and **B.** Target onset. Electrodes with significant gamma responses following the relevant trigger outlined in blue. Red crosses indicate reference electrodes.
(PDF)

S6 Fig Overlapping frequencies of beta and gamma activity in M1 and M2. **A.** Example choice-aligned spectra from M1 (blue) and M2 (red) showing peaks in the beta (15–29 Hz) low gamma (31–45 Hz) and high gamma (55–100 Hz) frequency bands. **B.** Scatter plot showing the peak frequency and amplitude detected in the beta, low and high gamma frequency bands for both M1 and M2. **C.** Summary of the peaks detected for both M1 and M2. Vertical lines denote the mean frequency of peaks in the beta, low and high gamma bands for M1 (blue) and M2 (red), respectively. Note the overlap of peak frequency of activity observed in both animals.
(PDF)

S7 Fig Choice-aligned spectrograms by abstract rule. Choice-aligned spectrograms of LFP activity recorded from FPC split by block (color and shape) and average across all recording sessions from both animals (left and middle). Statistical spectrogram showing the difference of color – shape. No significant differences survived cluster correction.
(PDF)

S8 Fig GLM analysis of choice-aligned activity in FPC. A. Example regressors derived from a single behavioral session input to the GLM analysis. Regressors include reward (black), the estimated value of the current (red) and the other abstract rule (cyan) and the trial-by-trial difference in both values, current rule delta (blue) and counterfactual rule delta (purple). **B.** The mean correlation between all five regressors averaged over all sessions from both animals. **C.** Spectrograms showing the Results from the GLM for two contrasts: the delta of the current, and the delta of the counterfactual rules.
(PDF)

S9 Fig Summary of the relationship between LFP activity in FPC and counterfactual and reward values for M1 and M2. Mean parameter estimates obtained from GLM analyses for beta (15–30 Hz), low gamma (30–45 Hz), and high gamma activity (55–100 Hz). Parameter estimates for counterfactual (light gray) and reward (dark gray) shown for both M1 and M2 separately. The data underlying this figure can be found via the following https://doi.org/10.12751/g-node.knk883.
(PDF)

S10 Fig Location and electrode groupings of stimulated electrodes. **A.** Array maps of the two arrays implanted in FPC, with the 6 which were stimulated shown in cyan (and numbered). **B.** The two protocols for the HF stimulation (cyan) and control stimulation (gray), with the first pulses labeled in each instance. **C.** Examples of the electrode groupings which were stimulated on each pulse. Electrode groupings were selected pseudorandomly from all possible permutations. Electrode pairings for each pulse were consistent across the two stimulation pairings.
(PDF)

S11 Fig Stimulation aligned behavioral analysis. **A.** Example schematic (*left panel*) showing the high-frequency stimulation protocol (cyan), and the probability of animals performing a correct trial and of making a rule error and a distractor error (aligned to the stimulated trial for both the high-frequency stimulation and nonstimulated blocks. **B.** Example schematic (*left panel*) showing the control frequency stimulation protocol (gray), and the probability of animals performing a correct trial and of making a rule error and a distractor error aligned to the stimulated trial for both the high-frequency stimulation and nonstimulated blocks.
(PDF)

S12 Fig Scatter plots showing the relationship between Explorative and Perseverative Errors for nonstimulated sessions (top left). For nonstimulated and stimulated blocks from HF-stimulation sessions (middle, left, and right, respectively) and for nonstimulated and stimulated blocks from control stimulation sessions (lower, left, and right, respectively).
(PDF)

S13 Fig Trial-by-trial spectral analysis showing the difference between the field potential in FPC following (A) control stimulation (B) nonstimulated blocks within the same sessions. Data shown are 400 ms periods of choice aligned **(B)** or inter-trial interval activity **(A)** from the 10 trials preceding and 10 trials following the poststimulation block change. Significant differences determined using one-sample *t* tests (see Materials and methods), thresholded and cluster corrected at *p* < 0.05. Spectrums showing the average change in LFP power following both stimulation protocols, for the 5 trials preblock change (red) and 7 trials postblock change shown on the right (gray). Spectra show mean ±SEM.
(PDF)

S14 Fig The full width half max (FWHM) of Morlet wavelets used to decompose LFP activity recorded from FPC. The FWHM in ms plotted against the central frequency of the wavelets (*left*) and the FWHM converted to frequency domain, again plotted against the central frequency of the wavelet (*right*).
(PDF)

## Abbreviations

AIC: Akaike Information Criterion
BL: Bayesian learner
E.E: explorative error
FPC: frontopolar cortex
LFPs: local field potentials
LPFC: lateral prefrontal cortex
NHPs: nonhuman primates
P.E.: perseverative error
RL: reinforcement learning
WCST: Wisconsin Card Sort Task
