# Supplementary figures and images for: Transient frontopolar cortex stimulation induces prolonged disruption to counterfactual processing
Source: PLoS Biol. 2025 Nov 18;23(11):e3003495. doi: 10.1371/journal.pbio.3003495 (PMC12674556; doi:10.1371/journal.pbio.3003495)

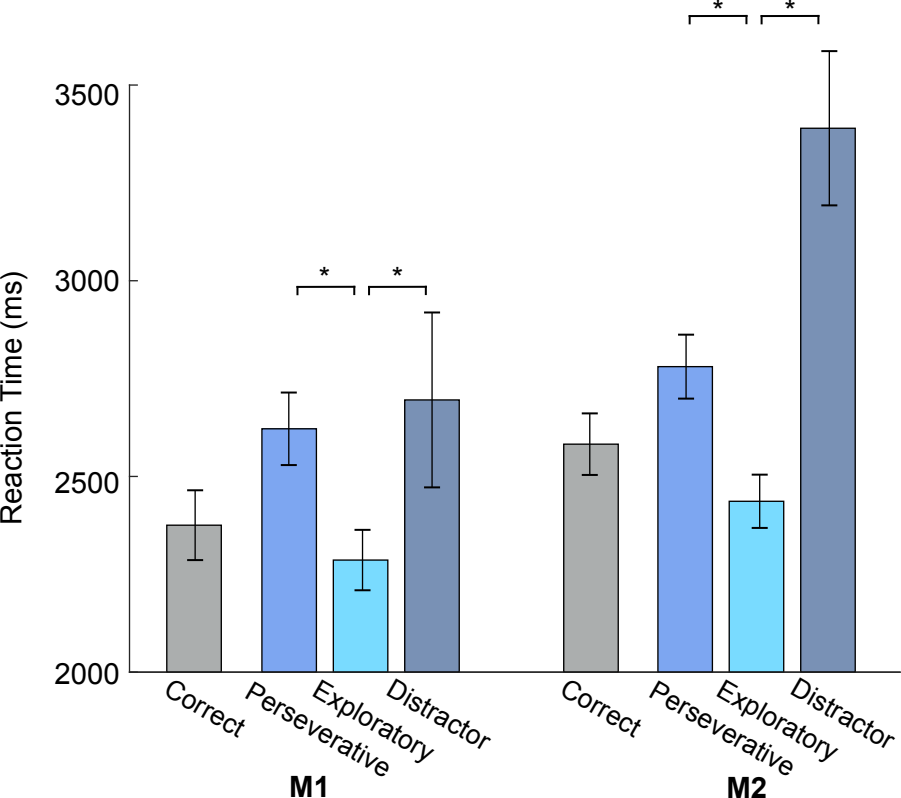

Supplement: S1 Fig — Bar charts showing the reaction times for monkey 1 and 2 for correct (gray) trials, as well as the three possible error outcomes. Rule errors (where the animals chose the incorrect rule) we split into two categories “perseverative errors” made by the animals over the first 7 trials of the block (blue) and “exploratory errors” made over the final 7 trials of the block (cyan). Distractor errors were made when the animals chose the target which didn’t correspond to either rule shown. (blue gray). Significant differences between the mean reaction times were tested with posthoc Bonferroni corrected t tests. The data underlying this figure can be found via the following https://doi.org/10.12751/g-node.knk883. (PDF) [file pbio.3003495.s001.pdf]

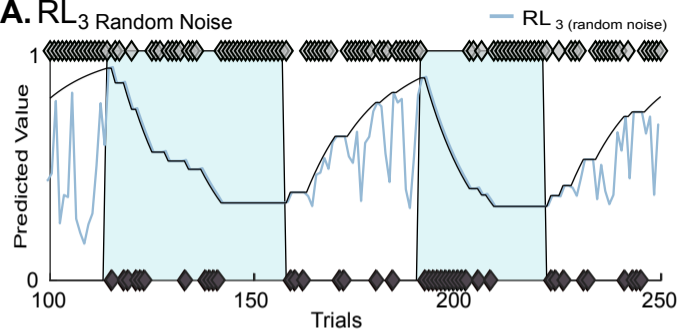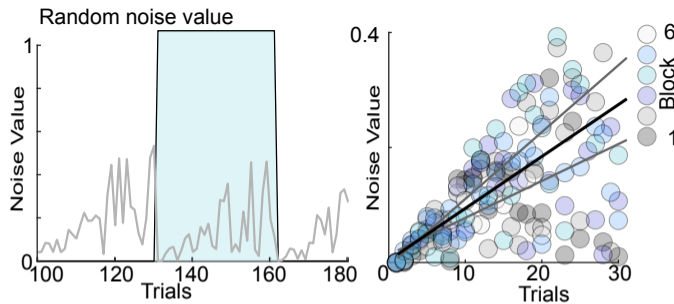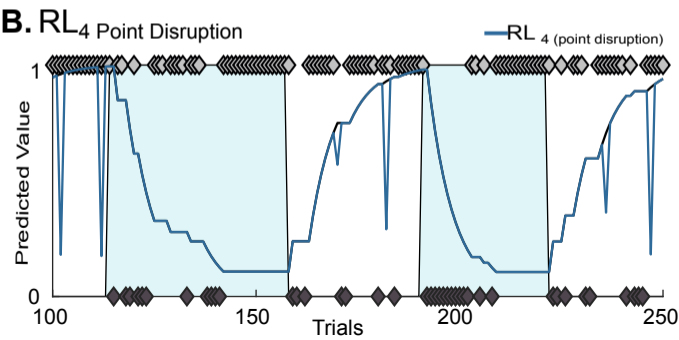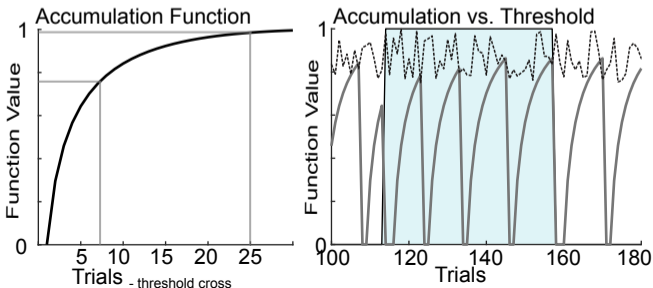

Supplement: S2 Fig — A. RL3 Random noise, values for shape and color predicted by RL3 were modified by addition of random noise to reflect decreased ability to remember the correct rule. Random noise increased in strength during the block. Example of the values of color predicted by RL3 (blue) after modification with randomly generated noise (left) with values obtained by RL1 shown for comparison (black). Correct trials denoted by light gray diamonds. Examples of the random noise component shown for 80 trials and all blocks of a single session (right). Mean random noise value ±SEM denoted with black and gray, respectively. B. RL4 point disruption, values for shape and color switched for a single trial to reflect animals momentarily assigning value on a single trial. Example of the values for color obtained from a single session by RL3 shown in blue with values for RL1 in black (left). The probability of the values assigned to color and shape switching increased after a block change until crossing a pseudo-random threshold (right, see Materials and methods). The upper and lower bounds of the threshold, and the accumulation function are shown in gray and black, respectively. Example of the pseudo-random threshold (black) and accumulation function (gray) shown for 80 trials. (PDF) [file pbio.3003495.s002.pdf]

**A**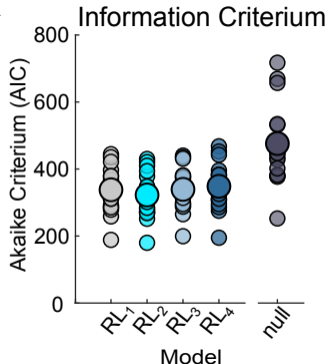**B**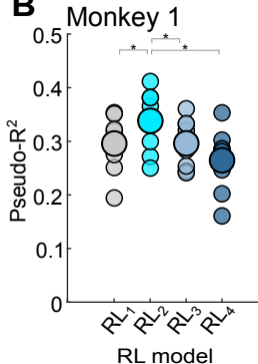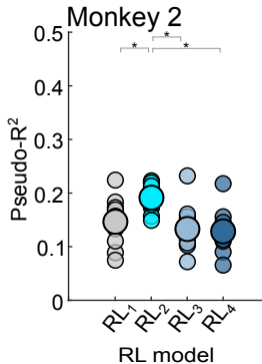

Supplement: S3 Fig — A. Akaike Information Criterium (AIC) used to calculate Mcfadden’s pseudo r2 shown for all four RL models against the null hypothesis, that animals chose randomly between the stimuli on each trial. B. Analysis of the pseudo r2 values obtained for all four RL models shown for both M1 and M2 separately. RL2 chosen + unchosen (light blue) provided a significantly better fit than the other three models in both animals. Asterisk denotes significant differences between model fit at p < 0.05). The data underlying this figure can be found via the following https://doi.org/10.12751/g-node.knk883. (PDF) [file pbio.3003495.s003.pdf]

A

Monkey M1

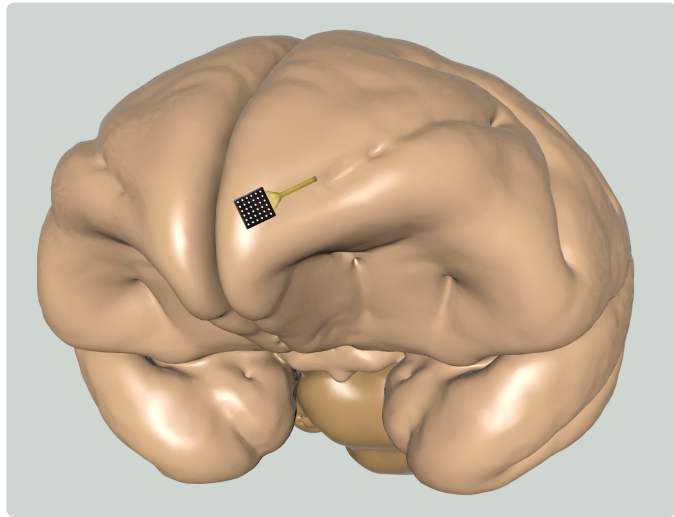

Monkey M2

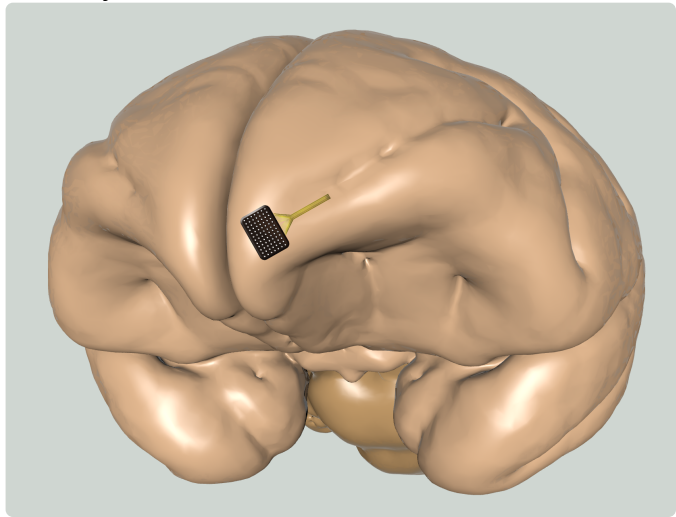

Supplement: S4 Fig — A. Diagrams showing the location of FPC arrays in M1 and M2 confirmed by postmortem examination of both animals’ brains. Note the difference in array size (32-channel and 64-channel arrays for M1 and M2, respectively). (PDF) [file pbio.3003495.s004.pdf]

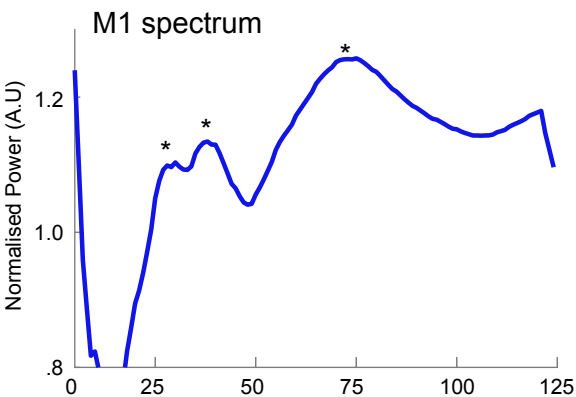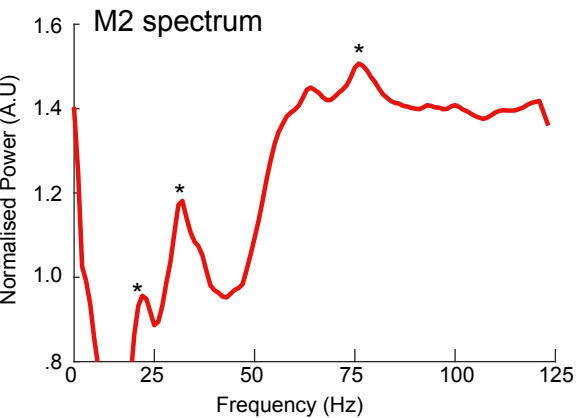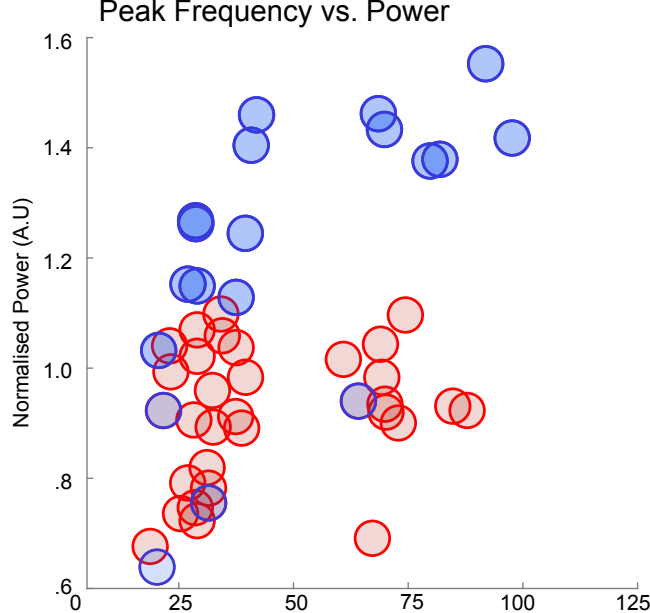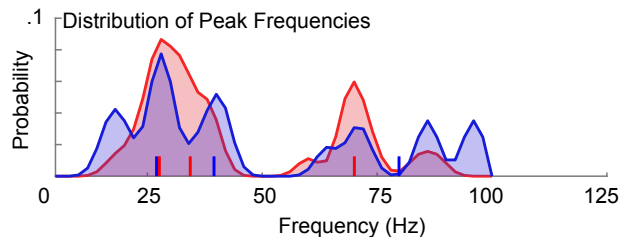

Supplement: S6 Fig — A. Example choice-aligned spectra from M1 (blue) and M2 (red) showing peaks in the beta (15–29 Hz) low gamma (31–45 Hz) and high gamma (55–100 Hz) frequency bands. B. Scatter plot showing the peak frequency and amplitude detected in the beta, low and high gamma frequency bands for both M1 and M2. C. Summary of the peaks detected for both M1 and M2. Vertical lines denote the mean frequency of peaks in the beta, low and high gamma bands for M1 (blue) and M2 (red), respectively. Note the overlap of peak frequency of activity observed in both animals. (PDF) [file pbio.3003495.s006.pdf]

**Colour**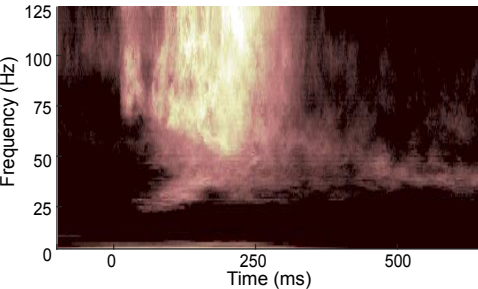**Shape**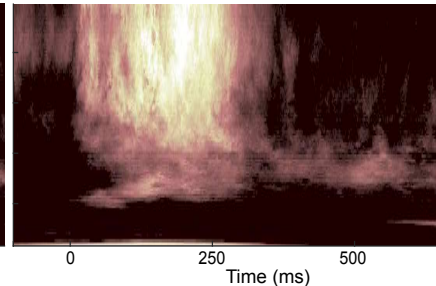**Contrast: Color-Shape**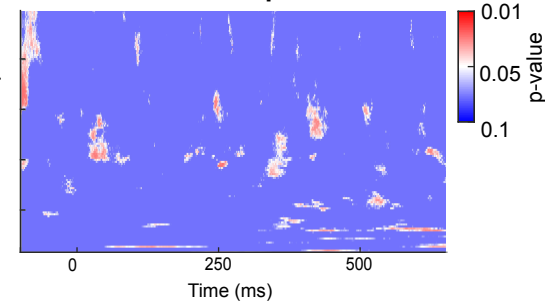

Supplement: S7 Fig — Choice-aligned spectrograms of LFP activity recorded from FPC split by block (color and shape) and average across all recording sessions from both animals (left and middle). Statistical spectrogram showing the difference of color – shape. No significant differences survived cluster correction. (PDF) [file pbio.3003495.s007.pdf]

### A Example Regressors

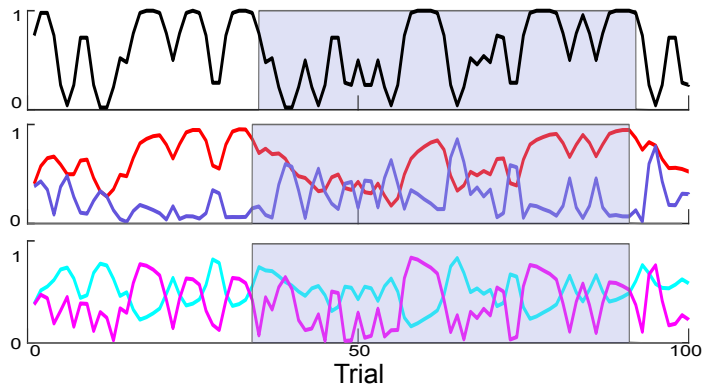

### B

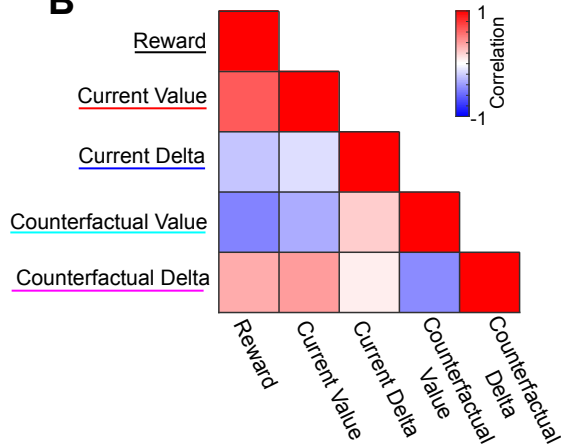

### C Contrast: Delta of current rule

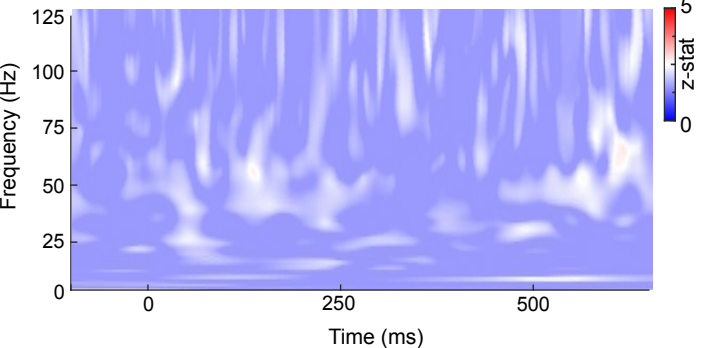

### Contrast: Delta of Other Rule

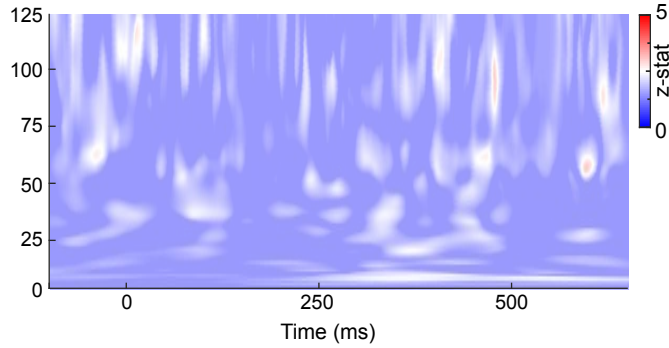

Supplement: S8 Fig — A. Example regressors derived from a single behavioral session input to the GLM analysis. Regressors include reward (black), the estimated value of the current (red) and the other abstract rule (cyan) and the trial-by-trial difference in both values, current rule delta (blue) and counterfactual rule delta (purple). B. The mean correlation between all five regressors averaged over all sessions from both animals. C. Spectrograms showing the Results from the GLM for two contrasts: the delta of the current, and the delta of the counterfactual rules. (PDF) [file pbio.3003495.s008.pdf]

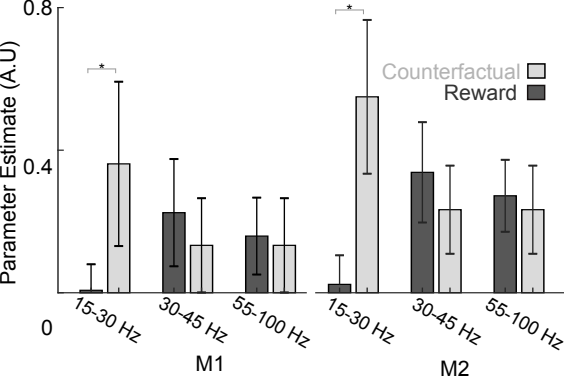

Supplement: S9 Fig — Mean parameter estimates obtained from GLM analyses for beta (15–30 Hz), low gamma (30–45 Hz), and high gamma activity (55–100 Hz). Parameter estimates for counterfactual (light gray) and reward (dark gray) shown for both M1 and M2 separately. The data underlying this figure can be found via the following https://doi.org/10.12751/g-node.knk883. (PDF) [file pbio.3003495.s009.pdf]

## A. High frequency stimulation

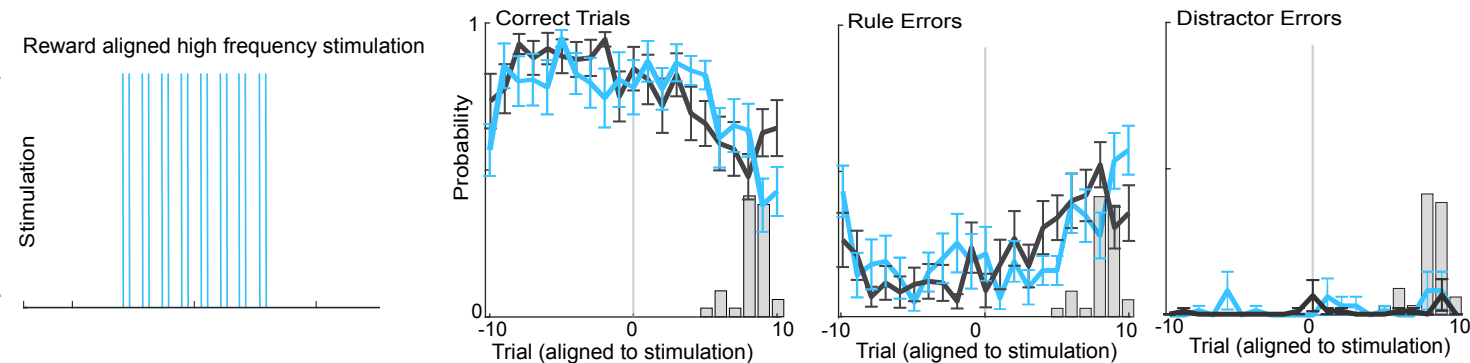

## B. Control stimulation

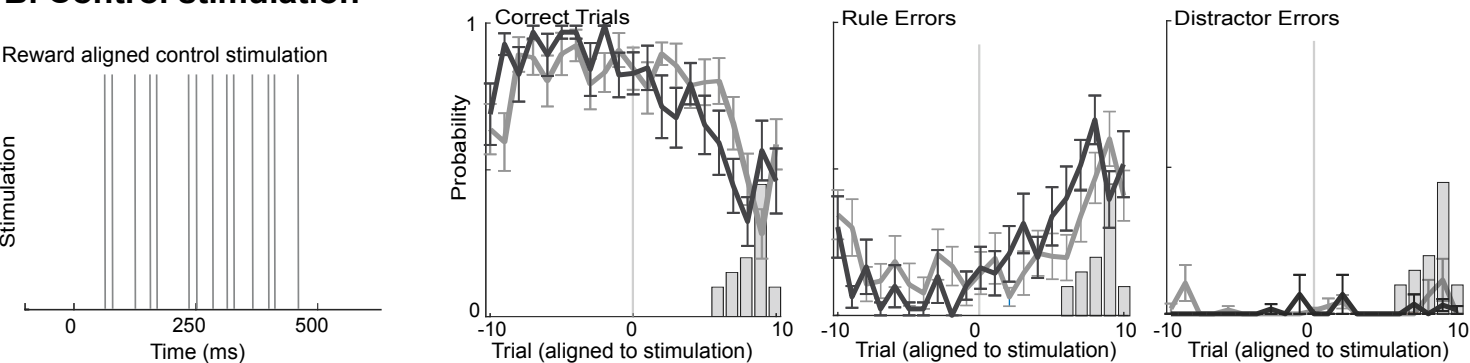

Supplement: S11 Fig — A. Example schematic (left panel) showing the high-frequency stimulation protocol (cyan), and the probability of animals performing a correct trial and of making a rule error and a distractor error (aligned to the stimulated trial for both the high-frequency stimulation and nonstimulated blocks. B. Example schematic (left panel) showing the control frequency stimulation protocol (gray), and the probability of animals performing a correct trial and of making a rule error and a distractor error aligned to the stimulated trial for both the high-frequency stimulation and nonstimulated blocks. (PDF) [file pbio.3003495.s011.pdf]

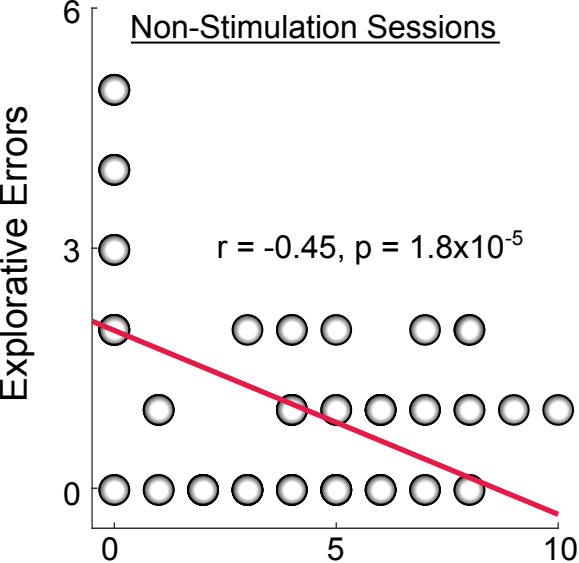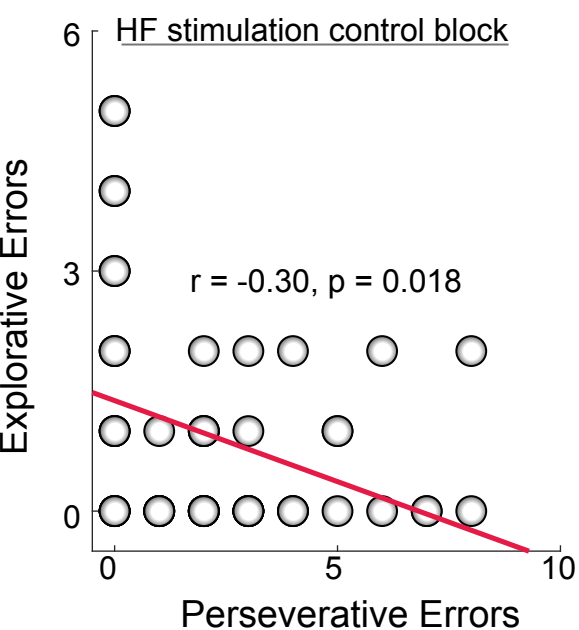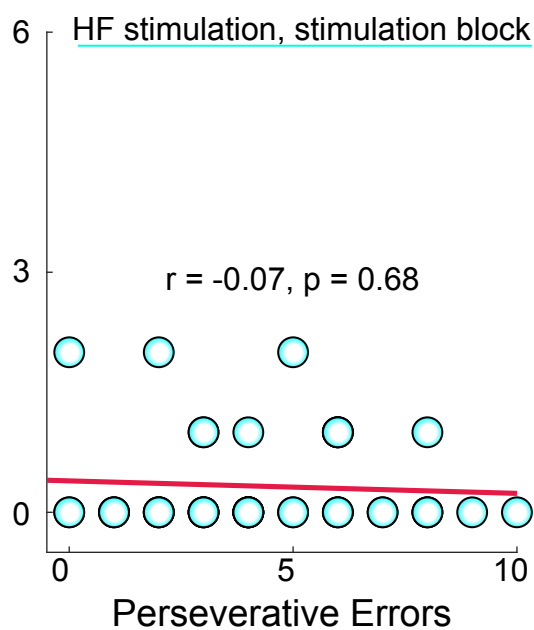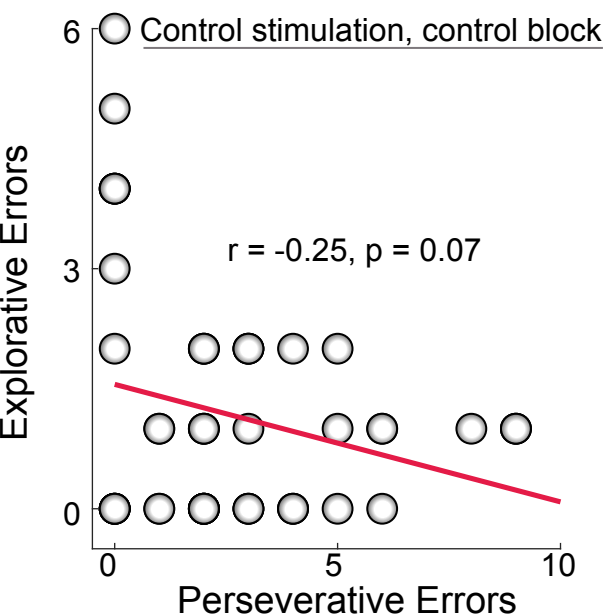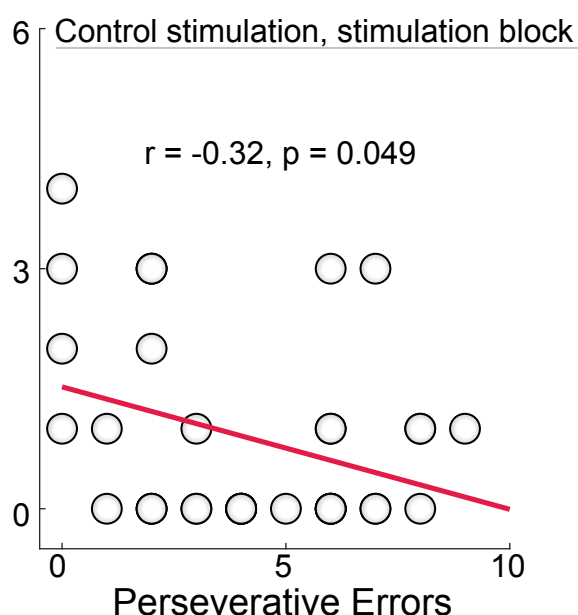

Supplement: S12 Fig — For nonstimulated and stimulated blocks from HF-stimulation sessions (middle, left, and right, respectively) and for nonstimulated and stimulated blocks from control stimulation sessions (lower, left, and right, respectively). (PDF) [file pbio.3003495.s012.pdf]

## A High frequency stimulation, inter-trial

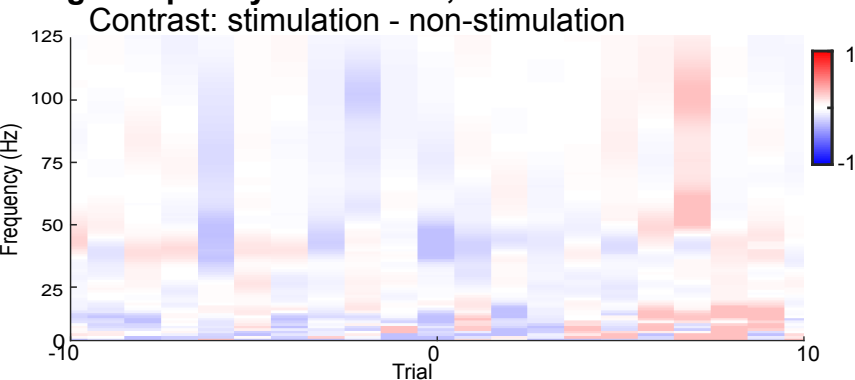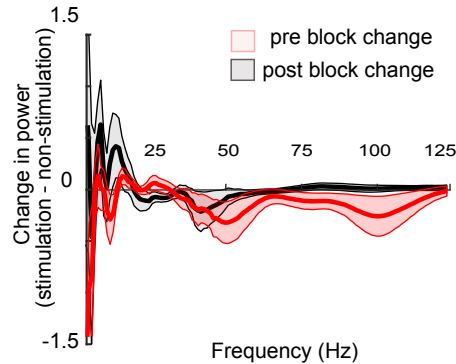

## B Control stimulation, choice-aligned

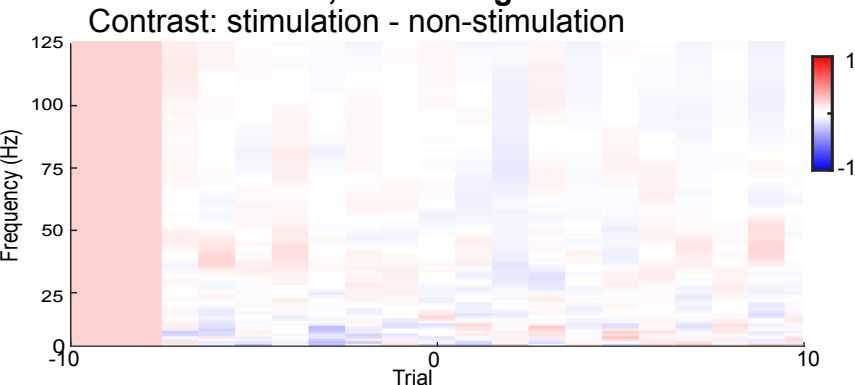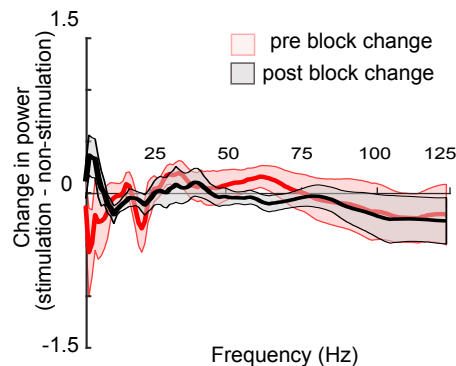

Supplement: S13 Fig — Data shown are 400 ms periods of choice aligned (B) or inter-trial interval activity (A) from the 10 trials preceding and 10 trials following the poststimulation block change. Significant differences determined using one-sample t tests (see Materials and methods), thresholded and cluster corrected at p < 0.05. Spectrums showing the average change in LFP power following both stimulation protocols, for the 5 trials preblock change (red) and 7 trials postblock change shown on the right (gray). Spectra show mean ±SEM. (PDF) [file pbio.3003495.s013.pdf]

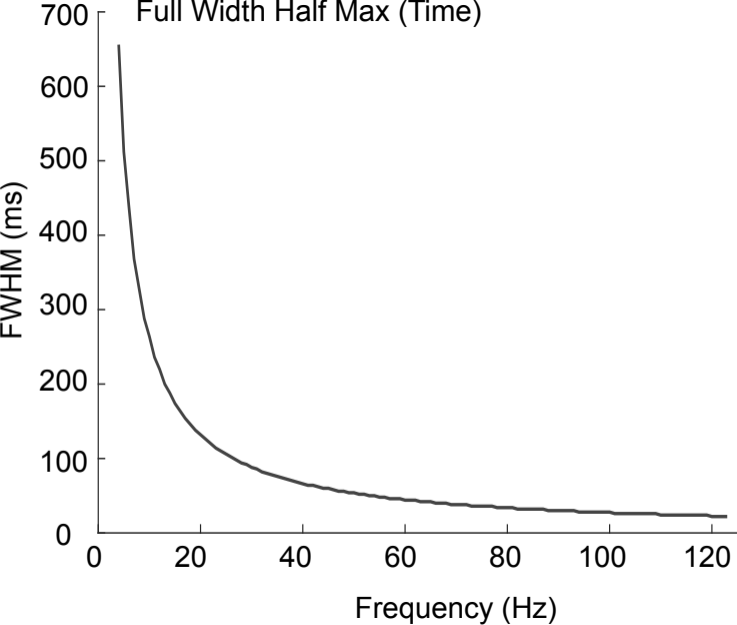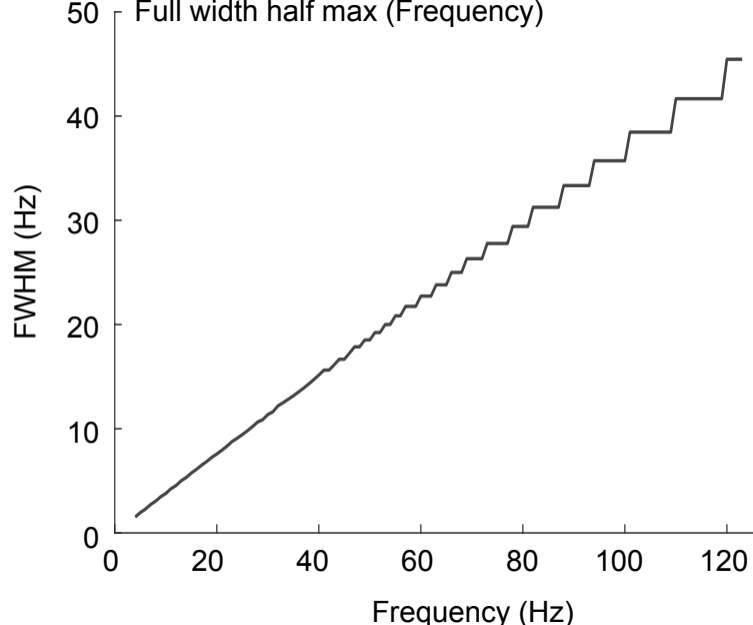

Supplement: S14 Fig — The FWHM in ms plotted against the central frequency of the wavelets (left) and the FWHM converted to frequency domain, again plotted against the central frequency of the wavelet (right). (PDF) [file pbio.3003495.s014.pdf]
