# Supplementary material for: Transient frontopolar cortex stimulation induces prolonged disruption to counterfactual processing
Source: PLoS Biol. 2025 Nov 18;23(11):e3003495. doi: 10.1371/journal.pbio.3003495 (PMC12674556; doi:10.1371/journal.pbio.3003495)
Supplement: S5 Fig — Mean spectrograms calculated from electrodes in FPC showing the absence of gamma activity (left panel) and maps showing the limited response in gamma activity observed in arrays implanted in M1 and M2 (right panels) aligned to A. Cue and B. Target onset. Electrodes with significant gamma responses following the relevant trigger outlined in blue. Red crosses indicate reference electrodes. (PDF) [file pbio.3003495.s005.pdf]

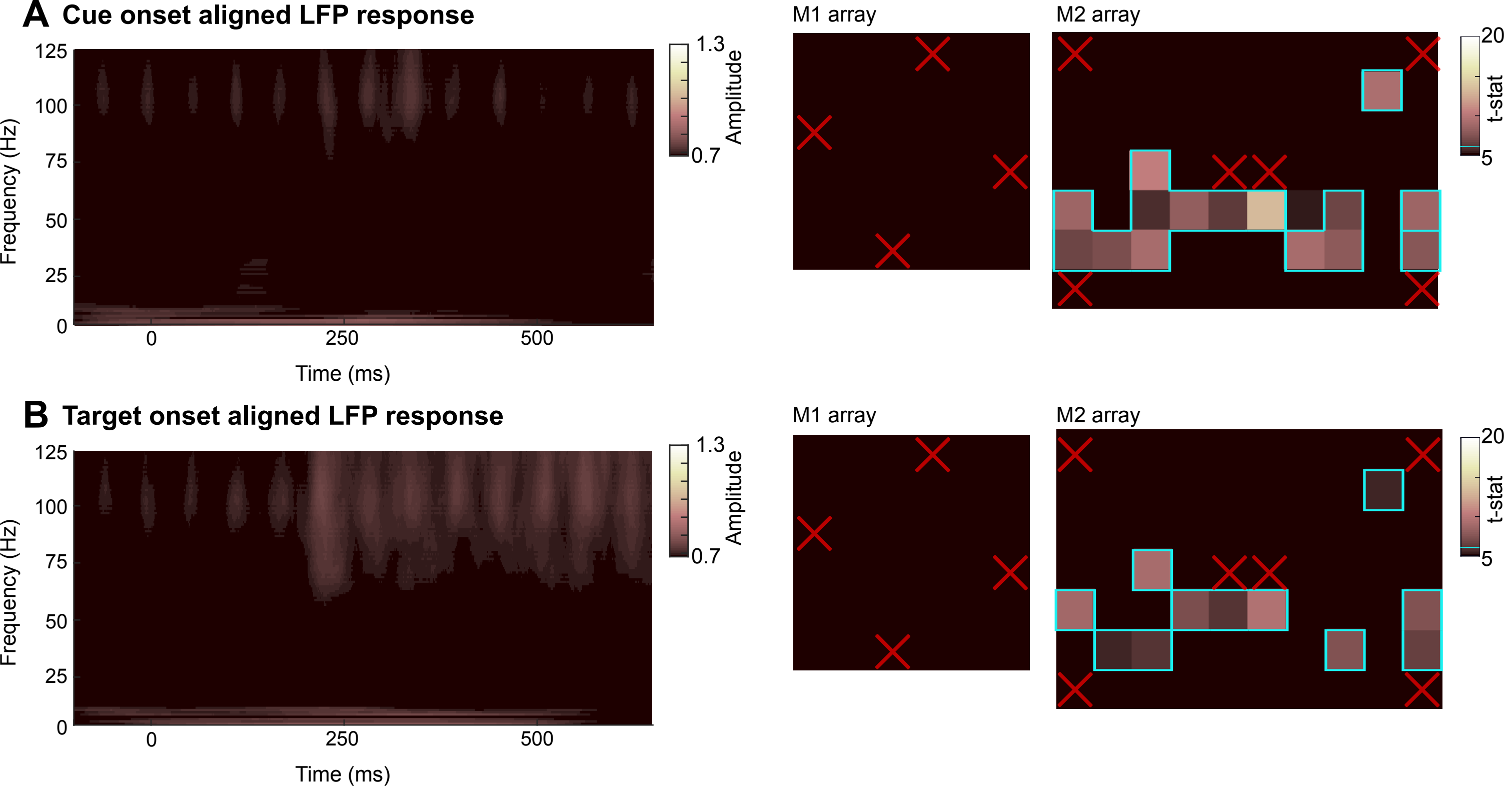
A Cue onset aligned LFP response
Frequency (Hz)
125
100
75
50
25
0
0
250
500
Time (ms)
1.3
0.7
Amplitude
M1 array
M2 array
20
5
t-stat
B Target onset aligned LFP response
Frequency (Hz)
125
100
75
50
25
0
0
250
500
Time (ms)
1.3
0.7
Amplitude
M1 array
M2 array
20
5
t-stat
