# Supplementary material for: Transient frontopolar cortex stimulation induces prolonged disruption to counterfactual processing
Source: PLoS Biol. 2025 Nov 18;23(11):e3003495. doi: 10.1371/journal.pbio.3003495 (PMC12674556; doi:10.1371/journal.pbio.3003495)
Supplement: S10 Fig — A. Array maps of the two arrays implanted in FPC, with the 6 which were stimulated shown in cyan (and numbered). B. The two protocols for the HF stimulation (cyan) and control stimulation (gray), with the first pulses labeled in each instance. C. Examples of the electrode groupings which were stimulated on each pulse. Electrode groupings were selected pseudorandomly from all possible permutations. Electrode pairings for each pulse were consistent across the two stimulation pairings. (PDF) [file pbio.3003495.s010.pdf]

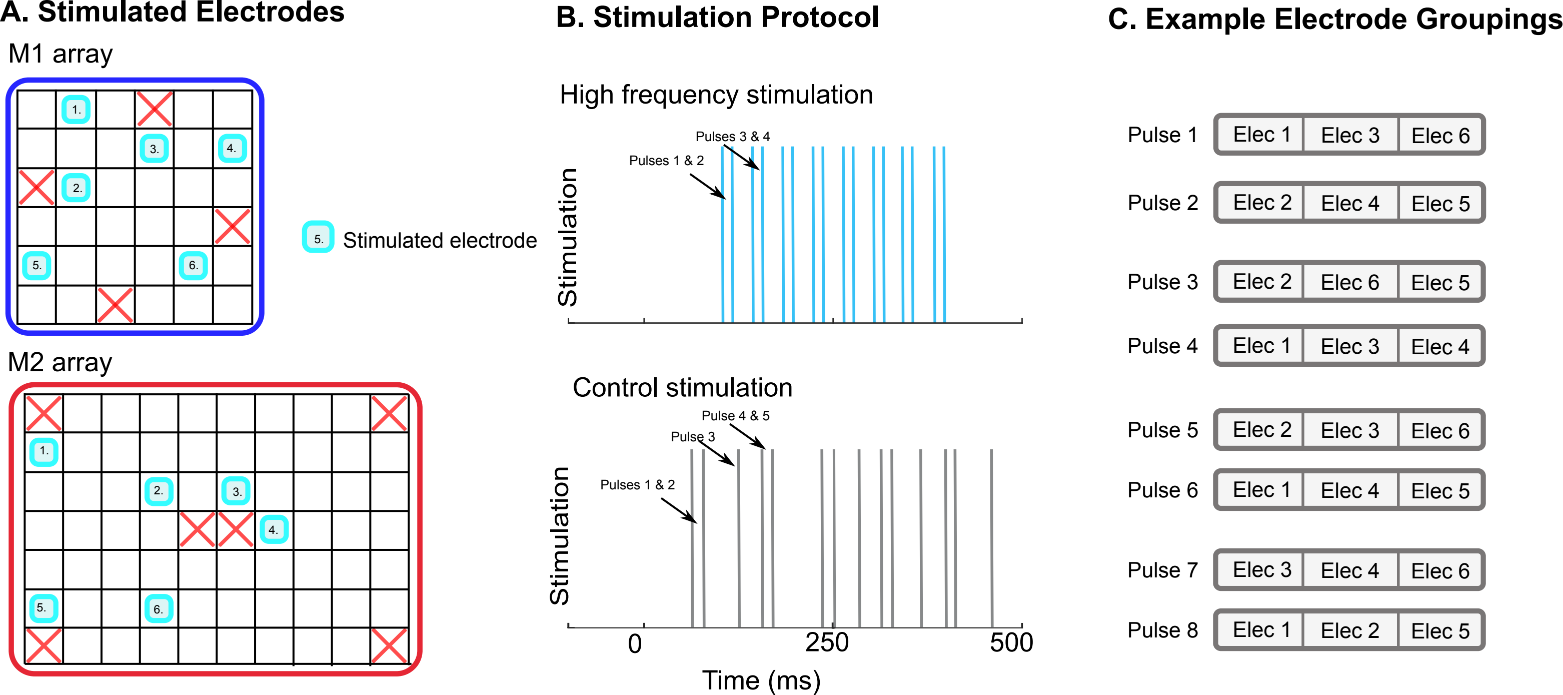

A. Stimulated Electrodes
M1 array
1.
3.
4.
2.
5.
6.
5.
Stimulated electrode
M2 array
1.
2.
3.
4.
5.
6.
B. Stimulation Protocol
High frequency stimulation
Pulses 3 & 4
Pulses 1 & 2
Stimulation
Control stimulation
Pulse 4 & 5
Pulse 3
Pulses 1 & 2
Stimulation
0
250
500
Time (ms)
C. Example Electrode Groupings
Pulse 1 Elec 1 Elec 3 Elec 6
Pulse 2 Elec 2 Elec 4 Elec 5
Pulse 3 Elec 2 Elec 6 Elec 5
Pulse 4 Elec 1 Elec 3 Elec 4
Pulse 5 Elec 2 Elec 3 Elec 6
Pulse 6 Elec 1 Elec 4 Elec 5
Pulse 7 Elec 3 Elec 4 Elec 6
Pulse 8 Elec 1 Elec 2 Elec 5
